# Enhancing the potential of phenomic and genomic prediction in winter wheat breeding using high-throughput phenotyping and deep learning

**DOI:** 10.3389/fpls.2024.1410249

**Published:** 2024-05-30

**Authors:** Swas Kaushal, Harsimardeep S. Gill, Mohammad Maruf Billah, Shahid Nawaz Khan, Jyotirmoy Halder, Amy Bernardo, Paul St. Amand, Guihua Bai, Karl Glover, Maitiniyazi Maimaitijiang, Sunish K. Sehgal

**Affiliations:** ^1^ Department of Agronomy, Horticulture and Plant Science, South Dakota State University, Brookings, SD, United States; ^2^ Department of Geography and Geospatial Sciences, Geospatial Sciences Center of Excellence, South Dakota State University, Brookings, SD, United States; ^3^ Hard Winter Wheat Genetics Research Unit, USDA-ARS, Manhattan, KS, United States

**Keywords:** wheat, high-throughput phenotyping (HTP) based traits, deep learning, phenomic prediction, deep neural network, multi-trait genomic selection

## Abstract

Integrating high-throughput phenotyping (HTP) based traits into phenomic and genomic selection (GS) can accelerate the breeding of high-yielding and climate-resilient wheat cultivars. In this study, we explored the applicability of Unmanned Aerial Vehicles (UAV)-assisted HTP combined with deep learning (DL) for the phenomic or multi-trait (MT) genomic prediction of grain yield (GY), test weight (TW), and grain protein content (GPC) in winter wheat. Significant correlations were observed between agronomic traits and HTP-based traits across different growth stages of winter wheat. Using a deep neural network (DNN) model, HTP-based phenomic predictions showed robust prediction accuracies for GY, TW, and GPC for a single location with R^2^ of 0.71, 0.62, and 0.49, respectively. Further prediction accuracies increased (R^2^ of 0.76, 0.64, and 0.75) for GY, TW, and GPC, respectively when advanced breeding lines from multi-locations were used in the DNN model. Prediction accuracies for GY varied across growth stages, with the highest accuracy at the Feekes 11 (Milky ripe) stage. Furthermore, forward prediction of GY in preliminary breeding lines using DNN trained on multi-location data from advanced breeding lines improved the prediction accuracy by 32% compared to single-location data. Next, we evaluated the potential of incorporating HTP-based traits in multi-trait genomic selection (MT-GS) models in the prediction of GY, TW, and GPC. MT-GS, models including UAV data-based anthocyanin reflectance index (ARI), green chlorophyll index (GCI), and ratio vegetation index 2 (RVI_2) as covariates demonstrated higher predictive ability (0.40, 0.40, and 0.37, respectively) as compared to single-trait model (0.23) for GY. Overall, this study demonstrates the potential of integrating HTP traits into DL-based phenomic or MT-GS models for enhancing breeding efficiency.

## 1 Introduction

In a world of finite resources, climate variability, and increasing population, ensuring food security has emerged as a critical challenge. To meet the rising global food demand, crop production needs to increase between 59–98% by 2050 compared to 2005 (Valin et al., 2014), translating to annual yield gains up to 2.4% for grain crops (Ray et al., 2013). However, genetic gains for yield in wheat (*Triticum aestivum* L.) are currently estimated to be less than 1% per year, significantly lower than the 2.4% target (Reynolds et al., 2012; Ray et al., 2013; Abberton et al., 2016).

Grain yield (GY) in wheat is a complex trait controlled by many genes and influenced by the interactions between genes and the environment (Heffner et al., 2011; Narjesi et al., 2015). Phenotyping plays a crucial role in breeding methods to enhance genetic gain in crops, however, it is time-consuming and expensive. Accurate prediction of yield and other complex traits across different growing cycles, environments, and breeding lines can significantly enhance the efficiency of the breeding program. However, the complex interplay between various factors makes such predictions challenging. Two promising strategies, high-throughput-phenotyping (HTP)-assisted phenomic selection and genomic selection (GS) have been explored with each having its unique advantages and limitations.

During the last decade, several HTP approaches have been developed to address the high labor and time costs associated with traditional phenotyping (White and Conley, 2013; Araus and Cairns, 2014). HTP platforms hold promise in linking the genotype to phenotype by increasing the data collection capacity (beyond visual range, repeated measures, and vast data handling), enhancing measurement precision, and minimizing the time required to evaluate large plant populations in fields. Further, HTP-assisted phenomic prediction incorporates field plot environmental conditions directly, which is not explicitly explored in standard GS models that rely on adjusted means estimated from the same plot data (Adak et al., 2022; Togninalli et al., 2023). Additionally, HTP can be performed across various growth stages and environments, drastically increasing phenotypic data to improve the selection accuracy (Lopes and Reynolds, 2012; Singh et al., 2016). HTP systems using various cameras and sensors have been implemented to measure simpler traits in wheat breeding programs, such as vegetation indices (VIs), growth rate, plant height, and disease resistance (Stewart et al., 2016; Jimenez-Berni et al., 2018; Khan et al., 2018; Rincent et al., 2018). Recently, HTP via Unmanned Aerial Vehicles (UAVs) is also becoming popular in modern breeding programs, generating large datasets of canopy spectral reflectance information (i.e., VIs). These VIs serve as UAV-HTP-based traits, providing an integrated measurement of canopy structure and photosynthetic activity based on the amount of light reflected off the crop canopy (Huete et al., 2002; Reynolds and Langridge, 2016; Krause et al., 2020).

Numerous statistical and machine-learning (ML)-based regression methods, including Multiple Linear Regression (MLR) (Jin et al., 2017), Partial Least Squares Regression (PLSR) (Rischbeck et al., 2016), Random Forest Regression (RFR) (Aghighi et al., 2018), and Support Vector Regression (SVR) (Kuwata and Shibasaki, 2015) have been applied to predict crop yields using HTP-based traits with variable accuracy. Deep Learning (DL), a subfield of ML, utilizes complex neural network architectures with multiple hidden layers. This allows DL models to achieve significantly higher accuracies in various tasks across different domains as compared to traditional ML methods. Techniques such as fully connected feedforward Deep Neural Network (DNN), Convolutional Neural Network (CNN), and Recurrent Neural Network (RNN) (LeCun et al., 2015; Schmidhuber, 2015; Ball et al., 2017; Cai et al., 2018; Sidike et al., 2019) have shown remarkable promise in solving both regression and classification problems. Recently, several studies have leveraged DL for prediction of different traits in various crops through the utilization of high-throughput plant phenotyping images as input. These studies utilized DL models to extract spectral features from leaf reflectance for disease scoring (Nagasubramanian et al., 2019; Picon et al., 2019), wheat spike segmentation and counting (Misra et al., 2020), and identification of Quantitative Trait Loci (QTLs) associated with root architecture traits (Pound et al., 2017). Although DNN has demonstrated promising results in other areas of plant phenotyping (Fieuzal et al., 2017; Pound et al., 2017; Misra et al., 2020), their potential in yield prediction in winter wheat remains unexplored. This presents a significant opportunity to leverage the power of DL for accurate and robust crop yield predictions across trials, breeding generations, and environments, potentially surpassing the capabilities of traditional statistical methods.

On the other hand, GS offers a promising approach for predicting the genomic estimated breeding value (GEBV) of lines utilizing genome-wide marker information (Meuwissen et al., 2001; Jannink et al., 2010). It has the potential to accelerate genetic gain by increasing selection intensity, accuracy and shortening the breeding cycle time. GS coupling with next-generation sequencing technology has been applied to several quantitative traits in wheat, including GY (Heffner et al., 2011; Poland et al., 2012; Sun et al., 2019; Gill et al., 2021), disease resistance (Rutkoski et al., 2012, 2014; Juliana et al., 2019; Zhang et al., 2022), and nutritional quality (Heffner et al., 2011; Manickavelu et al., 2017; Gill et al., 2023). Traditionally, most of the commonly used GS models have been single-trait (ST) models, which include phenotypic information about primary traits only, such as GY, test weight (TW), grain protein content (GPC), end-use quality attributes, or disease resistance, which are of main interest to the plant breeder. These single-trait genomic selection (ST-GS) models do not take advantage of the correlation between the primary trait of interest and secondary traits. Recently, multi-trait genomic selection (MT-GS) models that utilize the power of correlated HTP-based traits (Anche et al., 2020) have shown encouraging results. Improvement in prediction accuracies for traits with lower heritability has been observed by including correlated traits in the MT-GS model (Jia and Jannink, 2012). The incorporation of HTP-based traits and canopy temperature in the MT-GS models improved the prediction accuracy (PA) for GY in wheat by as much as 50% compared to the ST-GS model (Crain et al., 2018). These findings show that the incorporation of HTP traits improves the performance of GS models. These indirect estimates for selection are of great value in early generations of a breeding cycle when there’s insufficient seed for measuring quantitative traits or conducting multi-environment trials. Therefore, the prediction of quantitative traits at an early stage using HTP and genome-wide markers may improve selection accuracy.

In the present study, we aim to (1) evaluate the efficacy of a DNN model for phenomic prediction of GY, TW, and GPC using UAV assisted HTP-based traits while determining the optimal growth stage for collecting these traits in winter wheat, (2) evaluate the potential of forward GY prediction using HTP-based traits, and (3) evaluate the potential of HTP-based traits as covariates in the MT-GS models in prediction of complex agronomic traits, including GY in winter wheat breeding program.

## 2 Materials and methods

### 2.1 Experimental design and trait measurement

**Figure 1:**
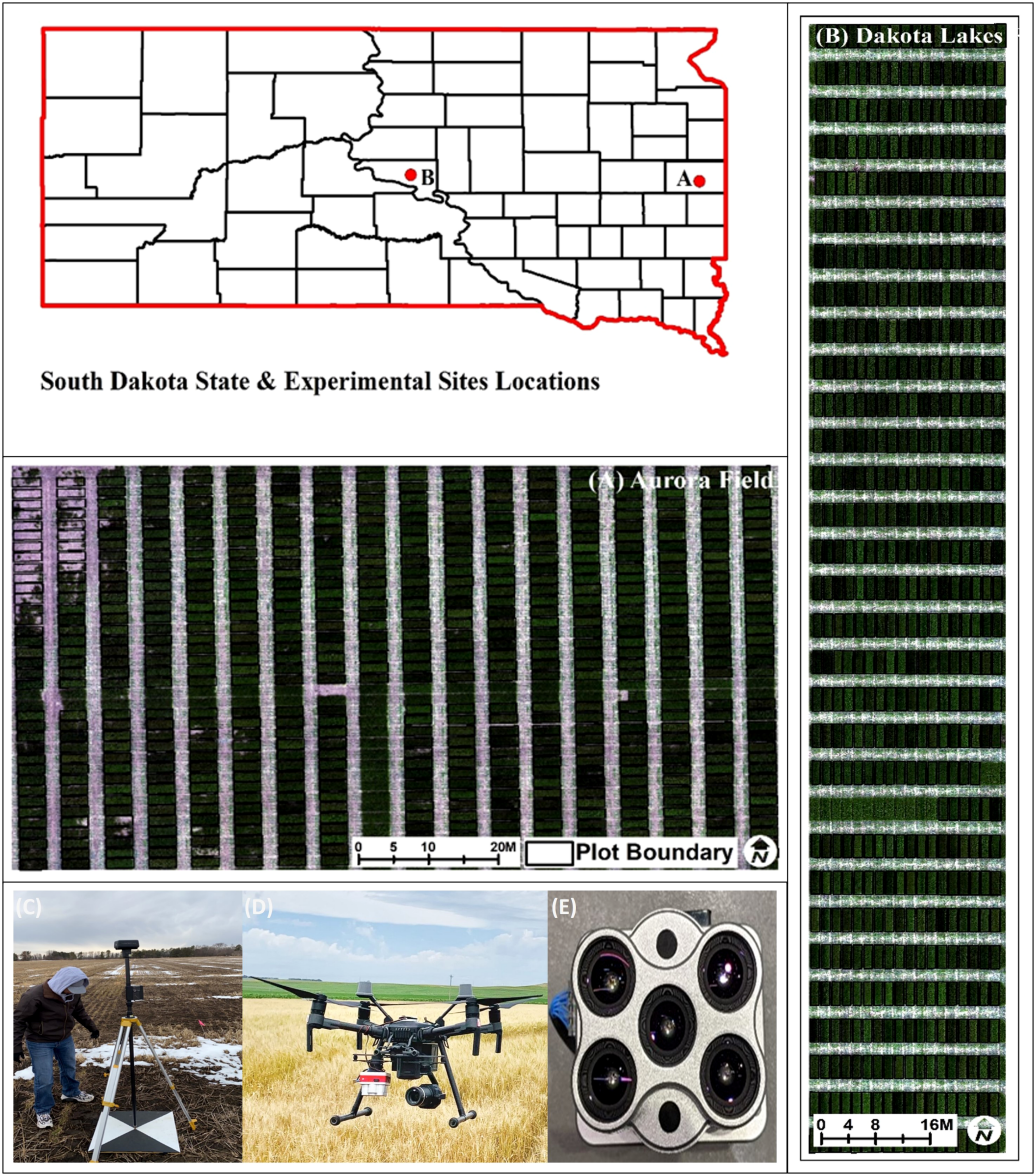
Testing site location map and UAV platform and sensor used in this study **(A)** Aurora Farm, Brookings (Brookings County, SD) and **(B)** Dakota Lakes Research Farm, Pierre (Huges County). Orthomosaic images created from drone-captured data are displayed for both sites. Also pictured are the tools used for data collection: **(C)** ground control points (GCPs) for image georeferencing, **(D)** a drone (unmanned aerial vehicle or UAV), and **(E)** a multispectral camera. GCPs are reference markers that ensure accurate positioning of the captured images. Drones provide a platform for high-resolution aerial imaging and carry multispectral camera that capture data across multiple wavelengths of light, allowing for detailed analysis.

The experiment was conducted at two sites located in Brookings (Brookings, County) and Dakota Lakes Research Farm (Huges County) in South Dakota (**Figures 1A, B**) during the 2021–2022 growing season using a total of 752 winter wheat genotypes (**Table 1**). The genotypes included 162 breeding lines from Elite Yield Trials (ELITE) and Advanced Yield Trials (AYT) from the South Dakota State University (SDSU) winter wheat breeding program, along with well-adapted check cultivars. In addition to ELITE and AYT, 597 lines from Preliminary Yield Trials (PYT) comprising 590 breeding lines and 7 elite check varieties were used as a validation population for GY prediction (independent validation) using the optimized training population (**Table 1**). The ELITE and AYT trials were planted using a randomized complete block design with three and two replicates, respectively whereas PYT was evaluated in augmented design with repeated checks across 22 blocks. The experimental plots were planted under a no-till system and each test site consists of 1.5 meters wide and 4 meters long plots with seven rows spaced 20 cm apart with a seeding rate of 300 seeds m^−2^. Locally recommended field management practices were followed for proper growth and yield. GY (kg/ha) was determined for each individual plots after harvesting at maturity using a plot combine (Zurn, Westernhausen Germany). TW and GPC were measured using Infratec™ 1241 Grain Analyzer (FOSS North America, Eden Prairie, MN, United States). GY and GPC were adjusted to 13% moisture content equivalence.

**Table 1:** Number of plots and genotypes planted in three nurseries at two locations in 2021–2022 for phenomic prediction and the number of Unmanned Aerial Vehicle (UAV) flights conducted.

Location	Nursery	Number of Genotypes	Number of observational plots	Number of Flights
Brookings, SD	ELITE	36	108	4
	AYT	126	252	4
	PYT	590	784	4
Pierre, SD	ELITE	36	108	4
	AYT	126	252	4

### 2.2 UAV system and flight missions

This study utilized a UAV system consisting of a Matrice 210 RTK V2 platform equipped with a five-band multispectral camera, Micasense Altum-PT (Micasense, Seattle, USA) (**Figures 1D, E**). The camera captures multispectral images with 3.2 Megapixel resolution (2064 x 1544). The multispectral camera comes with a standard calibration panel for radiometric calibration, which was imaged on the ground immediately before or after each flight. Four UAV flights were conducted between April and June 2022, corresponding to the growth stages of Jointing (Feekes 6/Zadoks 31), Flag leaf (Feekes 8/Zadoks 37), Booting (Feekes 10/Zadoks 45), and Milky Ripe (Feekes 11/Zadoks 75). The corresponding ground sampling distance (GSD) is 1.35 cm/pixel for the multispectral images. The flight altitude for the UAV was set at 45.0 m with a forward overlap of 75% and a side overlap of 75%. Each flight was conducted with a speed of 4 m/s between 10:00 am and 2:00 pm under windless and clear-sky conditions to ensure optimal image quality.

The second-generation Downwelling Light Sensor (DLS) was mounted on top of the drone and connected to the camera. This sensor data is used for radiometric calibration during image processing, which corrects for fluctuations in light conditions during the drone’s flight. To enable accurate georeferencing along with geometric calibration, ground control points (GCPs) were set up in each field. These GCPs consisted of black and white cross-centered wooden boards (2x2 feet) placed evenly over the field (**Figure 1C**). The Global Positioning System (GPS) location of these GCPs was measured by a survey grade GNSS RTK GPS receiver (DJI), with centimeter accuracy in the X, Y, and Z directions.

### 2.3 UAV remote sensing data processing

**Figure 2:**
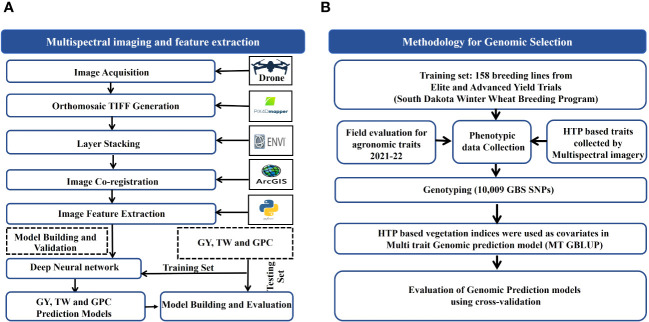
Schematic illustration of **(A)** Phenomic Selection (PS) for grain yield (GY), test weight (TW) and grain protein (GPC) and **(B)** Genomic Selection (GS) utilizing UAV-assisted High-Throughput Phenotyping (HTP)-based traits.

Raw multispectral images were orthorectified and mosaicked using Pix4Dmapper software (Pix4D, Lausanne, Switzerland) (Maimaitijiang et al., 2019). The geometric correction was performed during the orthomosaicking process in Pix4Dmapper using the GCPs established at the data collection sites. Pix4Dmapper conducted automatic radiometric calibration during the orthomosaicking process, utilizing calibration data gathered from the calibration panel. Additionally, ambient light variations were captured using the MicaSense DLS mounted atop the UAV platform. The UAV orthomosaiced image dataset was layer-stacked using ENVI software. Then, the layer-stacked images were co-registered in the ArcMap software (Ormsby, 2004) using GCPs (**Figure 2**). This ensures a common georeferenced extent for images of each acquisition date with WGS 1984 datum UTM Zone 14 N.

### 2.4 Feature extraction from imagery

Spectral features such as VIs were derived from the orthorectified multispectral images, utilizing average canopy reflectance values for each of the five bands of multispectral imagery at the plot level using Python programming. All these spectral features are illustrated in **Supplementary Table 1**.

### 2.5 Deep neural networks

**Figure 3:**
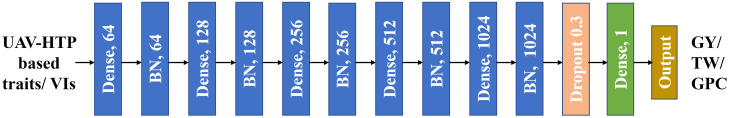
A schematic illustration of the Deep Neural Network (DNN) model architecture used for phenomic prediction in this study. The input consists of Unmanned Aerial Vehicle (UAV)-High Throughput Phenotyping (HTP)-based traits followed by five dense hidden layers, each containing 64, 128, 256, 512, and 1024 neurons, respectively. The activation function utilized is linear, and dropout with a rate of 0.3 is applied. The output of the model includes GY, GPC, and TW predictions. Batch normalization denoted as “BN”.

The DNN with multiple hidden layers apply nonlinear functions to the output of each hidden layer, enhancing their capability to learn intricate, nonlinear relationships between input and response variables (LeCun et al., 2015). These networks model complex, non-linear relationships between input variables (e.g., HTP based traits) and the output (e.g., GY). The DNN model used in our study is a feedforward fully connected deep neural network with multiple hidden layers (**Figure 3**). Rectified linear unit (ReLU) activation function was employed in fully connected layers, while linear activation function was utilized for regression (Glorot et al., 2011). Mean Squared Error (MSE) served as the loss function, and the Adam Optimizer was employed for weight updates during training. The DNN model was tuned with various hyperparameters, including learning rate, dropout rate, and batch size. The epoch parameter was explored across a range, including 50, 100, 150, 200, and 250, while batch size was varied between 16, 24, 32, and 64. The total plots were randomly split into training (70%) and testing (30%) sets. DNN models were trained with gradient-based optimization methods to minimize the loss function. For our study, we utilized Python, along with Matplotlib and Seaborn for visualization, and scikit-learn and TensorFlow for developing and training/testing prediction models (Hunter, 2007; Van Rossum, 2007; Abadi et al., 2016).

#### 2.5.1 Model evaluation

To evaluate the performance of the prediction model, we used the coefficient of determination (R^2^) Equation (1), root mean square error (RMSE) Equation (2), and relative root mean square error (RMSE%) Equation (3). y_i_ and ŷ_i_ refers to the measured and the predicted GY/GPC/TW, $\bar{\text{y}}$ is the mean of measured GY/GPC/TW, and n is the total number of samples in the testing set.


(1) $$R^{2}=1-\frac{\sum_{i=1}^{n}{\left(yi-\hat{y}\right)}^{2}}{\sum_{i=1}^{n}{\left(yi-\bar{y}\right)}^{2}}$$

(2) $$RMSE=\sqrt{\frac{\sum_{i=1}^{n}{\left(yi-\hat{y}\right)}^{2}}{n-1}}$$

(3) $$RMSE\%=\frac{RMSE}{\bar{y}}*100$$

To assess the robustness and transferability of the DNN model, we further evaluated its performance in predicting GY in 784 early-generation breeding (PYT) plots using model trained on 360 ELITE and AYT plots from a single location (Scenario 1) and 720 ELITE and AYT plots from multi-location (Scenario 2). Thus, determining how well the model generalizes to unseen data, a crucial aspect for real-world application in breeding programs.

### 2.6 Genotyping-by-sequencing

Fresh leaf tissues were obtained from each line to isolate DNA using a modified cetyl-trimethyl ammonium bromide (CTAB method) (Maguire et al., 1994). A genotyping-by-sequencing (GBS) library was constructed using the *Pst*I and *Msp*I restriction enzymes (Poland et al., 2012). The library was sequenced on NextSeq2000 sequencer (Illumina, San Diego, 5200 Illumina Way, USA) at the USDA Central Small Grain Genotyping Lab in Manhattan, KS, USA. Single-nucleotide polymorphism (SNP) variants were called using the GBS v2.0 SNP discovery pipeline in TASSEL v5.0 (Trait Analysis by aSSociation, Evolution, and Linkage) (Bradbury et al., 2007) and the Chinese Spring (CS) wheat genome RefSeq v2.1 (IWGSC, 2018; Zhu et al., 2021) as the reference. For quality control, SNPs with more than 30% missing genotypes, minor allele frequencies (MAF) below 5%, and those not mapped on any chromosome were excluded before imputation using BEAGLE v4.1 using default parameters except setting ‘ne = 12000’ (Browning and Browning, 2007), which generated 10,009 high-quality SNPs for further analysis.

### 2.7 Genomic prediction models and cross-validation

#### 2.7.1 Statistical analysis of the phenotype data

All the statistical analyses were performed using various libraries in R (R Core Team, R, 2021). For the experimental design, best linear unbiased estimates (BLUEs) for various traits were estimated using the model given in Equation 4:


(4) $$y_{ij}=\text{μ}+R_{i}+G_{j}+e_{ij}$$

where *y_ij_* is the trait of interest, µ refers to the overall mean, *R_i_* denotes the random effect of the i^th^ replicate, *G_j_* is the fixed effect of the j^th^ genotype, and *e_ij_* refers to the residual error effect of the i^th^ replication and j^th^ genotype. The broad-sense heritability (*H* ^2^) for agronomic and HTP-based traits was estimated by fitting the genotypic effect from the above equation as random, using the following formula given in Equation 5:


(5) $$H^{2}=\frac{\;\sigma_{g}^{2}}{\;\frac{\sigma_{g}^{2}+\sigma_{e}^{2}}{nRep}}$$

where $\sigma_{g}^{2}$ and $\sigma_{e}^{2}$ , are the genotype and error variance components, and nRep refers to the number of replicates. The above analysis was performed in ‘lme4’ package (Bates et al., 2015). The correlations among traits were estimated and visualized based on the BLUEs for each trait.

#### 2.7.2 Single-trait model

Standard genomic best linear unbiased prediction (GBLUP) was used as a baseline for comparison with MT-GS model. GBLUP employs a genomic relationship (G) matrix and the model for wheat lines (*i* = 1, 2, *…, n*) is given in Equation 6:


(6)  y=1µ + Zg + e

where **y** is the vector (n × 1) of BLUE values for each trait; µ is the overall mean; **1** is the vector of ones; **g** represents the genomic estimated breeding values with $g\;∼\;\text{N}\left(0,\;G\sigma_{g}^{2}\right)$ , where **G** is the genomic relationship matrix (VanRaden, 2008), $\sigma_{g}^{2}$ is the additive genetic variance and **e** is the vector of residual errors with $e\;∼\;\text{N}\left(0,\;\sigma_{e}^{2}\right)$ . The *p* biallelic centered and standardized markers (SNPs) are represented in incidence marker **Z** of order *n* x *p* (where *n* = 162) such that $G=\;\frac{XX'}{p}$ . The ST models were implemented with 5,000 burn-ins and 25,000 iterations of the Gibbs sampler in the R package BGLR (Pérez and de los Campos, 2014).

#### 2.7.3 Multi-trait model

A multivariate model was used to predict GY, GPC, and TW by including HTP-based traits (i.e., VIs) as secondary traits in the model. The MT model predicts primary traits using the secondary traits as shown in the Equation 7:


(7) $$\left[\begin{array}{c} {\text{y}}_{1}\\ \vdots \\ {\text{y}}_{\text{n}} \end{array}\right]=\left[\begin{array}{cc} \text{I} & 0\\ \vdots & \vdots \\ 0 & {\text{I}}_{\text{n}} \end{array}\right]\left[\begin{array}{c} {\text{μ}}_{1}\\ \vdots \\ {\text{μ}}_{n} \end{array}\right]+\left[\begin{array}{cc} \text{Z} & 0\\ \vdots & \vdots \\ 0 & {\text{Z}}_{\text{n}} \end{array}\right]\left[\begin{array}{c} {\text{g}}_{1}\\ \vdots \\ {\text{g}}_{\text{n}} \end{array}\right]+\left[\begin{array}{c} {\text{e}}_{1}\\ \vdots \\ {\text{e}}_{\text{n}} \end{array}\right]$$

where **y** is the n-dimensional vector of BLUEs for n traits, **I** and **Z** are the design matrices, *μt*, t = 1 … n, refers to trait intercepts of n traits, $\left[\overset{g_{1}}{\underset{g_{\text{n}}}{\vdots }}\right]\;$ are the predicted genetic values assumed to be distributed as ~MVN(0, **∑** ⊗ **G**) with **G** representing the genomic relationship matrix obtained following (VanRaden, 2008) and ⊗ refers to the Kronecker product of two matrices. The residuals of the MT model were assumed to be distributed as $\left[\overset{e_{1}}{\underset{e_{\text{n}}}{\vdots }}\right]\;$ ~MVN(0,**R** ⊗ **I**). The matrices **∑** and **R** are the variance-covariance matrices for the genetic and residual effects between traits, respectively, where **∑** was estimated as an unstructured variance-covariance matrix and R as a diagonal variance-covariance matrix. The MT GBLUP was implemented in the MTM package in R (Campos and Grüneberg, 2016) using the Gibbs sample algorithm with 5000 burn-ins and 15,000 iterations.

#### 2.7.4 Assessment of predictive ability

The predictive ability of GS models was estimated as Pearson correlation coefficient between GEBVs and observed phenotypes for the testing set of breeding lines. The ST GBLUP model with cross-validation scheme 1 (ST-CV1) was implemented for one trait at a time (**Supplementary Figure 1**). ST-CV1 assessed the ST model, random 80% lines for training (including both genotypic and phenotypic data) and the remaining 20% lines for validation (genotypic data only). The cross-validation process was repeated 100 times, and each iteration included different lines in the training and testing sets. The PA of the MT model was estimated to be used with cross-validation scheme 2 (MT-CV2) (Lado et al., 2018) (**Supplementary Figure 1**). In MT-CV2, lines were divided into sets of 80% for training and 20% for testing, and the procedure was repeated 50 times using random sets. The model was trained using the genotypic data and the phenotypic data of the primary trait for the training set, including the HTP traits for both the training and testing sets, to predict the primary trait in the testing set.

## 3 Results

### 3.1 Heritability and correlations analysis of HTP-based traits with agronomic traits

**Figure 4:**
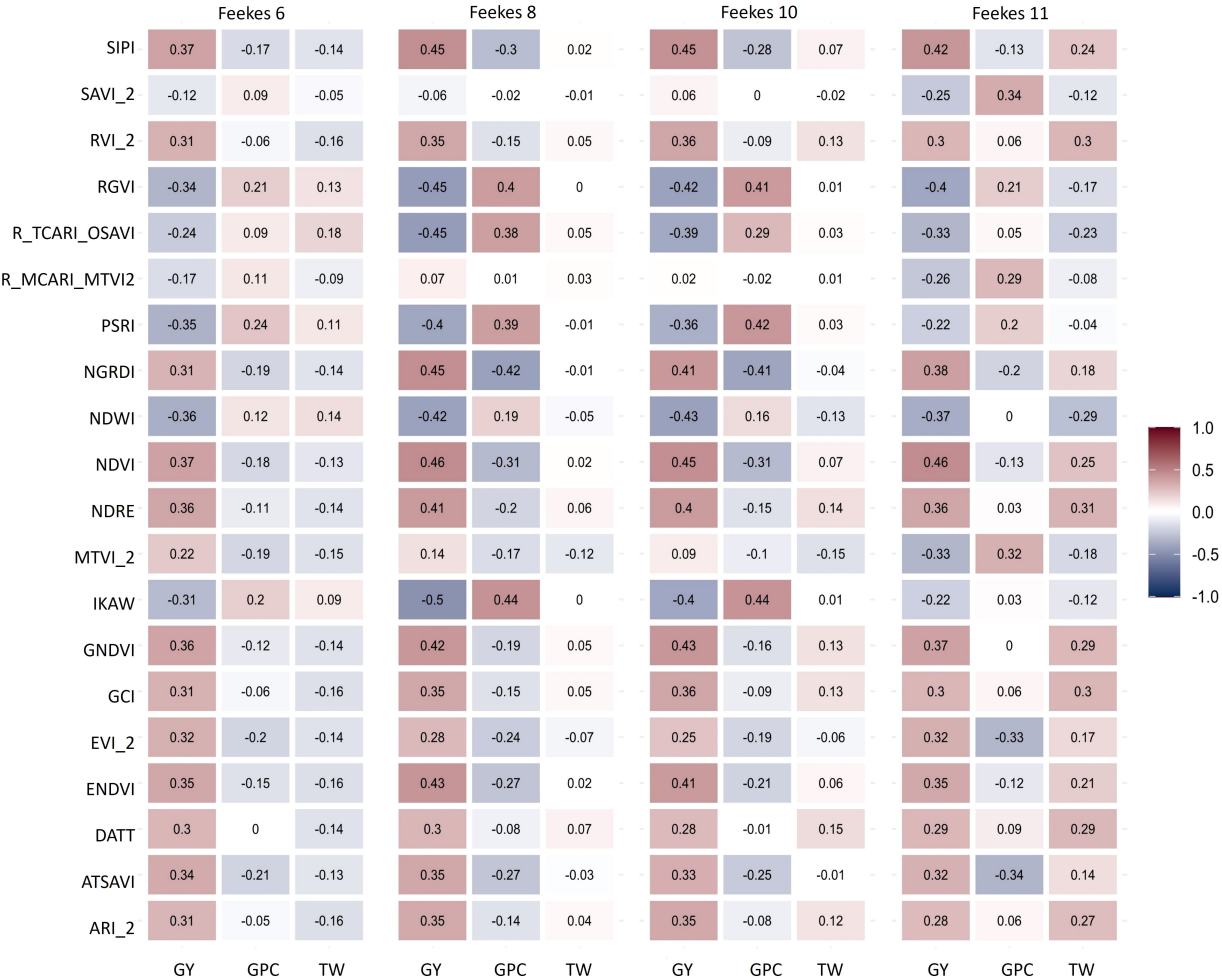
Correlation among High Throughput Phenotyping (HTP)-based traits from various growth stages (Feekes 6: Jointing, Feekes 8: Flag leaf, Feekes 10: Booting, Feekes 11: Milky ripe) and grain yield (GY), grain protein content (GPC), and test weight (TW) in hard winter wheat.

Broad-sense heritability for GY and GPC was lower as compared to TW (**Supplementary Table 2**), while heritability varied for various HTP-based traits (**Supplementary Figure 2**). Notably, HTP-based traits showed higher heritability during the later stages of crop growth, particularly at Feekes 10 and 11 (**Supplementary Figure 2**). Significant correlations were observed between primary traits (GY, TW, and GPC) and HTP-based traits, with these correlations varying across different growth stages or flights (**Figure 4**). This variation in correlations across growth stages allows us to leverage HTP-based traits for predicting primary traits. GY exhibited a stronger correlation with HTP-based traits during the Feekes 8 (Flag leaf) and Feekes 10 (Booting) stage, while TW showed a higher correlation during the Feekes 11 (Milky Ripe) stage (**Figure 4**). However, correlations were sparse or zero for GPC at Feekes 6 (Jointing stage) and for TW at the Feekes 10 and 11 stages. Examining the correlation of HTP-based traits with GY across different growth stages revealed significant associations. Notably, most HTP-based traits from later stages (Feekes 8, 10 and 11) displayed significant positive correlations with GY, while specific HTP-based traits like Plant Senescence Reflectance Index (PSRI), Kawashima index (IKAW), and Normalized Difference Water Index (NDWI) exhibited notable negative correlations across all growth stages.

### 3.2 Phenomic prediction using HTP-based traits and deep learning models

**Figure 5:**
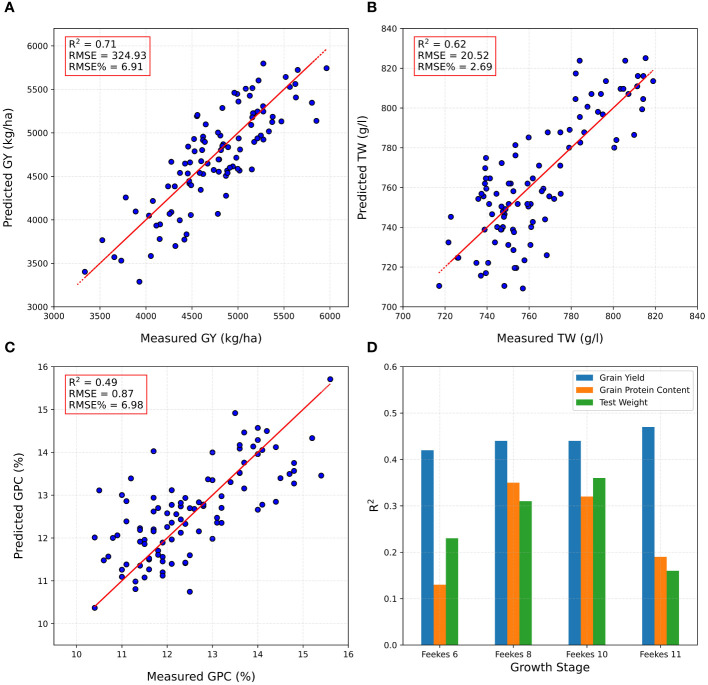
Phenomic predictions on the testing set/prediction set, using the DNN model developed using combined high throughput phenotyping traits (HTP)-based traits for **(A)** grain yield (GY), **(B)** test weight (TW), **(C)** grain protein content (GPC), and **(D)** prediction accuracy at a specific growth stage at Brookings location.

The complete set of plots from the ELITE and AYT were randomly split into a training set (70% of the ELITE and AYT plots), and the remaining 30% were used as a testing set (prediction set) to assess the performance of the DNN model developed using individual location (Brookings) and multi-locations (Brookings and Pierre). The DNN model was employed to predict GY, TW, and GPC using multitemporal HTP-based traits from all growth stages/flights in ELITE and AYT at one location. PA was assessed based on the RMSE% and R^2^ values. In the training set, the PA of the DNN model achieved R² values of 0.90 for GY, 0.85 for TW, and 0.79 for GPC (**Supplementary Figure 3**). Remarkably, in the testing set (prediction set), the highest R^2^ value achieved was 0.71 for GY prediction using all growth stages HTP-based traits, signifying the model’s robust performance in GY prediction (**Figure 5A**). However, for predicting TW and GPC, R^2^ values were slightly lower at 0.62 and 0.49, respectively, indicating varying prediction accuracies across these traits (**Figures 5B, C**). Additionally, RMSE% ranged from 2.69 to 6.98, underscoring the variance in PAs observed for these traits. The performance of DNN models was improved significantly when trained on a larger dataset. By combining HTP-based traits from ELITE and AYT trials of two locations (Brookings and Pierre) to train the DNN model, PA for the testing set further increased to 0.76 for GY, 0.64 for TW, and 0.75 for GPC (**Supplementary Figure 4**).

The DNN model exhibited varied predictive performance depending on the growth stages (Feekes 6, 8, 10, and 11) at which HTP-based traits were collected. For GY prediction in the testing set for ELITE and AYT at Brookings, the model achieved PA values with R^2^ ranging from 0.42 to 0.47. Notably, the Feekes 6 (Jointing) stage had the lowest PA (R^2^ = 0.42), while the Feekes 11 (Milky Ripe) stage highlighted the most robust performance, attaining the highest R^2^ value of 0.47 (**Figure 5D**). For TW predictions, the highest accuracy was attained during the Feekes 10 stage (R^2^ = 0.36), whereas the Feekes 11 stage showed the lowest PA (R^2^ = 0.13) (**Figure 5D**). Regarding GPC predictions, the highest accuracy was obtained at the Feekes 8 stage (R^2^ = 0.35), whereas the lowest PA was observed at the Feekes 6 stage (R^2^ = 0.13) (**Figure 5D**). These results highlight the differential predictive accuracies across growth stages for distinct traits, emphasizing the varying predictive capacities of the model at different developmental phases. Furthermore, they underscore the importance of selecting critical developmental stages to conduct HTP for optimal prediction.

### 3.3 Application of DNN models in forward prediction in a breeding pipeline

**Figure 6:**
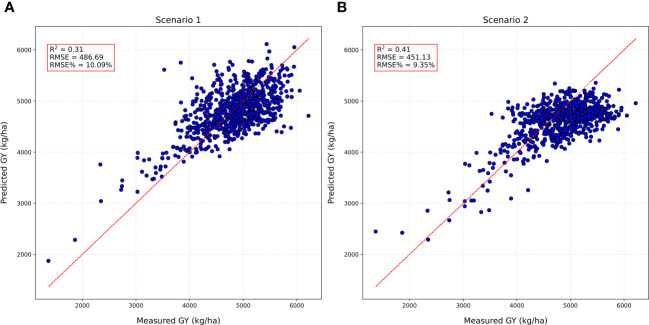
Model testing results of forward prediction for grain yield (GY) in preliminary yield trials (PYT) using DNN trained on high throughput phenotyping traits (HTP)-based traits from advanced breeding lines **(A)** Scenario 1: DNN model trained on (ELITE and AYT) at Brookings to predict GY in PYT at Brookings; **(B)** Scenario 2: DNN model trained on (ELITE and AYT) at Brookings and Pierre to predict GY in PYT at Brookings.

Next, we explored the potential of DNN models for forward prediction of GY in preliminary breeding nurseries. This approach involves training a DNN model on data from advanced breeding lines and then using it to predict GY in earlier breeding stages. We explored two distinct scenarios. In the first scenario (scenario 1), the DNN model was trained using HTP-based traits derived from four growth stages and GY of advanced breeding lines (ELITE and AYT) from a single location (Brookings). Subsequently, the model’s ability in forward prediction was evaluated by predicting the GY of 784 plots of PYT lines, also evaluated at Brookings. The model achieved an R^2^ of 0.31 and RMSE% = 10.08 for GY in PYT at Brookings (**Figure 6A**). This suggests that the model can make reasonably accurate predictions for lines in earlier breeding stages, even with HTP-based traits from limited samples from advanced breeding generations. In the second scenario (scenario 2) we investigated the impact of incorporating HTP-based trait samples from four growth stages from multiple locations. The DNN model was trained using HTP-based traits derived from four flights from the same ELITE and AYT but collected from two locations: Brookings and Pierre. A total of 720 plots were used for training. The model’s predictive capability was assessed by evaluating its ability to predict GY for 784 plots of PYT nursery at Brookings. The model achieved an R^2^ of 0.41 and an RMSE% of 9.35 for GY in PYT at Brookings (**Figure 6B**).

### 3.4 Genomic prediction

#### 3.4.1 Predictive ability of ST model

**Figure 7:**
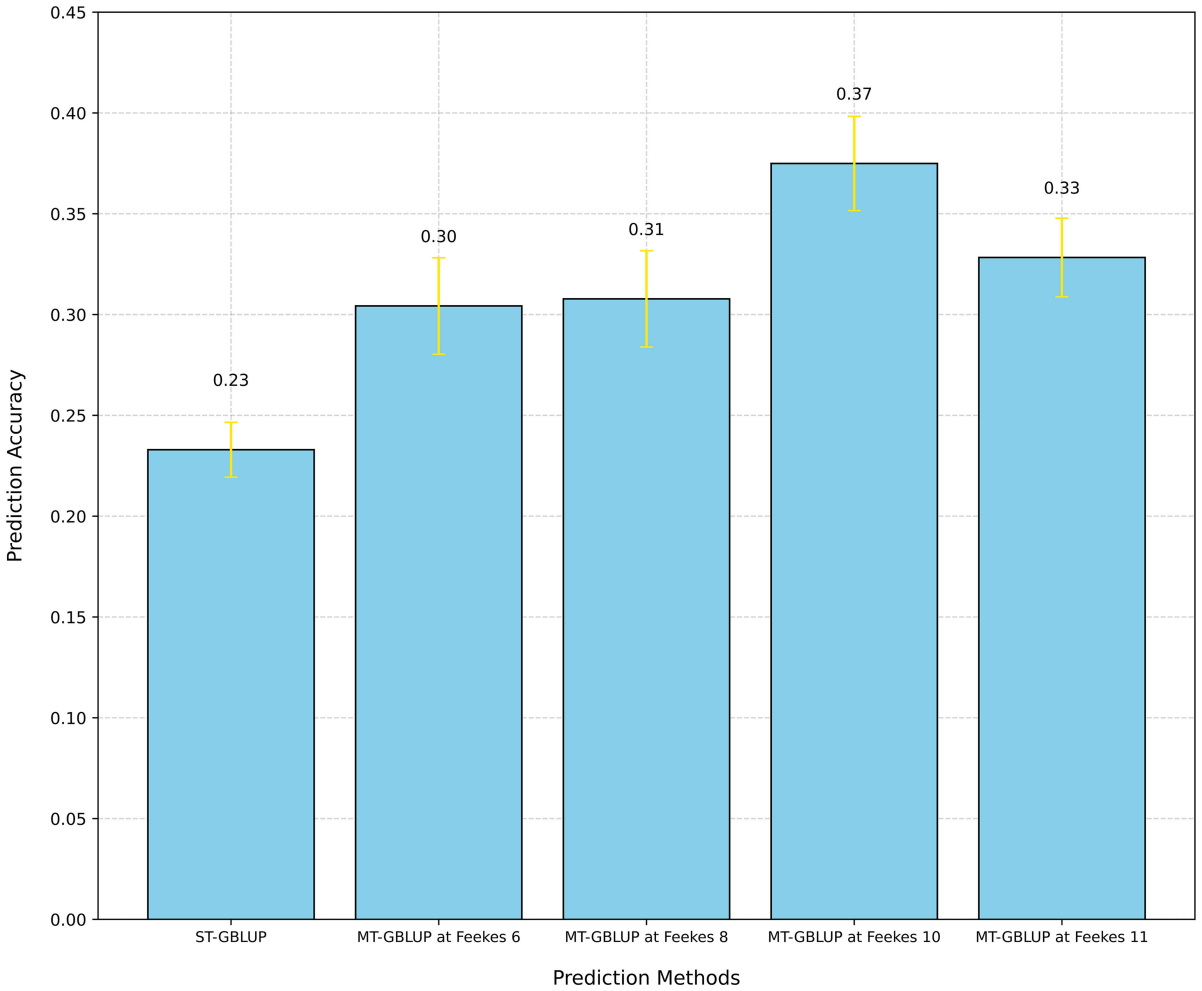
Comparison of prediction accuracy for grain yield (GY) using Single-Trait GBLUP (ST-GBLUP) with cross-validation scheme ST-CV1 and Multi-Trait GBLUP (MT-GBLUP) incorporating High-Throughput Phenotyping (HTP)-based traits with cross-validation scheme MT-CV2 across different growth stages (Feekes 6: Jointing, Feekes 8: Flag leaf, Feekes 10: Booting, Feekes 11: Milky ripe).

We used ST-GBLUP to assess the predictive ability of GY, TW, and GPC using the ST-CV1 scheme (**Supplementary Figure 1**). ST-GBLUP was selected as a baseline to compare with the MT-GBLUP models. We observed a PA of 0.23 for GY, 0.38 for TW, and 0.07 for GPC using ST-GBLUP (**Figure 7**; **Supplementary Figures 5**, **6**).

#### 3.4.2 Predictive ability of multi-trait model using HTP traits

**Figure 8:**
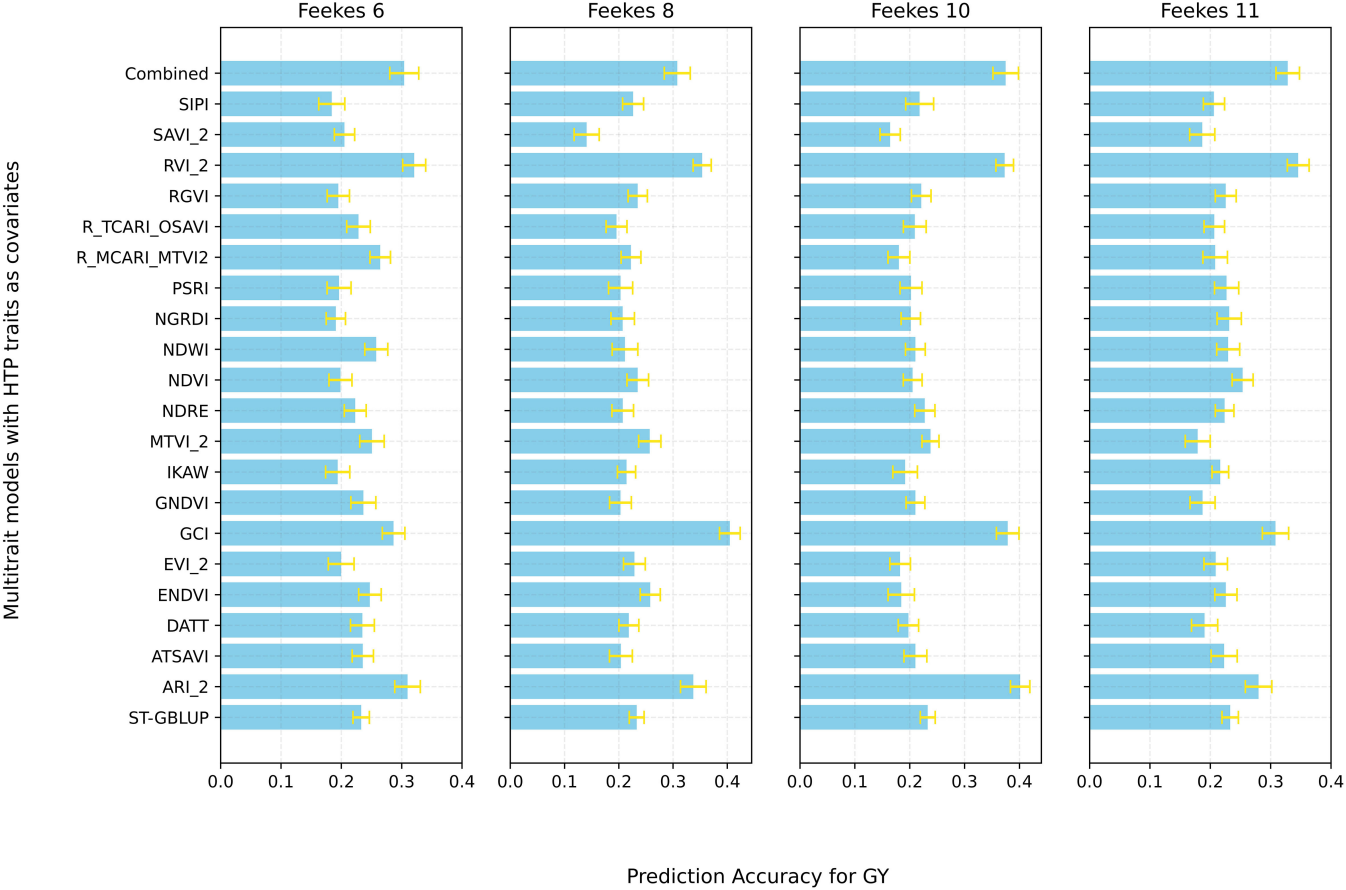
Prediction accuracy for grain Yield (GY) using Single-Trait GBLUP (ST-GBLUP) with cross-validation scheme ST-CV1 and Multi-Trait GBLUP (MT-GBLUP) incorporating High-Throughput Phenotyping (HTP)-based traits with cross-validation scheme MT-CV2 across different growth stages (Feekes 6: Jointing, Feekes 8: Flag leaf, Feekes 10: Booting, Feekes 11: Milky ripe). The *x-axis* presents respective prediction accuracy, and the *y-axis* presents MT models with different combinations of HTP-based traits as covariates and ST-GBLUP at the bottom as baseline for comparison.

We also employed the MT-model to evaluate the predictive results of GY, TW, and GPC using the MT-CV2 scheme (**Supplementary Figure 1**). First, all HTP-based traits (20 VIs) at specific growth stages were utilized as covariates in the MT model to predict the primary traits (GY, TW, and GPC). Significant improvement in PA were observed at specific growth stages for different traits, with Feekes 10 for GY (0.37), Feekes 6 for TW (0.40), and Feekes 8 for GPC (0.18) showing the highest PAs (**Figure 7**; **Supplementary Figures 5**, **6**). Additionally, individual assessments of each HTP-based trait within the MT model revealed a range of PA for GY, spanning from 0.14 to 0.40 (**Figure 8**). Feekes 8 (Flag leaf) exhibited the highest accuracy (0.40) in predicting GY when employing the Green Chlorophyll Index (GCI), while the lowest accuracy (0.14) was observed at Feekes 8 using Soil Adjusted Vegetation Index 2 (SAVI_2). Interestingly, among all HTP-based traits, certain VIs like GCI, Ratio Vegetation Index 2 (RVI_2), and Anthocyanin Reflectance Index 2 (ARI_2) displayed comparable or even better PAs compared to using all HTP-based traits together in the MT-GS model for GY.

For TW, the MT-model PA ranged from 0.34 to 0.40 (**Supplementary Figure 6**). The Feekes 11 (Milky ripe) stage demonstrated the highest accuracy (0.49) with RVI_2, whereas the Feekes 8 owed the lowest (0.40) when utilizing SAVI_2. Similar to GY predictions, specific HTP-based traits such as ARI_2, RVI_2, and Enhanced Normalized Difference Vegetation Index (ENDVI) achieved similar or even better PAs for TW in contrast to employing all HTP-based traits together in the MT-GS model (**Supplementary Figure 7**). Regarding GPC, the MT-GS model PAs varied from 0.06 to 0.18 (**Supplementary Figure 5**). The Feekes 8 stage presented the highest accuracy (R^2^ = 0.14) using RVI_2, while the lowest accuracy (0.01) was observed at the Feekes 8 stage with Modified Chlorophyll Absorption in Reflectance Index (MCARI) (**Supplementary Figure 8**). Analogous to GY and TW findings, certain HTP-based traits like EVI_2, Normalized Difference Vegetation Index (NDVI), and Transformed Chlorophyll Absorption in Reflectance Index - Optimized Soil-Adjusted Vegetation Index (TCARI_OSAVI) demonstrated comparable or superior PAs for GPC compared to the collective use of all HTP-based traits in the MT-GS model. Distribution of PA for GY, TW, and GPC varied across replicates for ST-GS with ST-CV1 and MT-GS with MT-CV2 incorporating HTP traits across each flight (**Supplementary Figures 9**, **10**, and **11**). This highlights the influence of cross-validation scheme and model choice on the PA of primary traits.

## 4 Discussion

In the present work, we evaluated the effectiveness of the DL-based DNN model for phenomic prediction of wheat agronomic traits (GY, TW, and GPC) using UAV-HTP-based traits under various scenarios. We also assessed the efficacy of incorporating HTP-based traits as covariates into MT genomic prediction models. The DNN method was chosen for its ability to manage high-dimensional data, such as multitemporal HTP-based traits collected across various growth stages. These HTP-based traits encompass a vast number of features, and the DNN method excels at automatically extracting complex relationships between these input features and the target traits, eliminating the need for manual feature engineering (Mishra et al., 2021). Additionally, the DNN method has shown superior PA compared to traditional ML methods for complex tasks like crop yield prediction (Khaki and Wang, 2019). Overall, the DNN model exhibited a wide range of performance in phenomic prediction of GY, TW, and GPC in advanced breeding lines. An impressive R^2^ value of 0.71 was achieved for GY, with acceptable accuracies for TW (R^2^ = 0.62) and GPC (R^2^ = 0.49) (**Figure 5**). These findings highlight the model’s varying effectiveness across different traits, potentially due to the complex genetic and environmental factors influencing each trait. The performance of the DNN model improved significantly when trained on a larger dataset. Combining HTP data from two locations boosted testing set PA for all traits: GY by 7%, TW by 3%, and GPC by a remarkable 53%. Overall, these findings highlight the importance of utilizing substantial and varied datasets when training DL methods for enhanced predictions. These results align with recent observations that found a prediction accuracy (PA) of 0.64 for grain yield (GY) prediction using multi-sensor data fusion in wheat (Fei et al., 2023). The observed variations in RMSE% further highlighted differences in PAs across the traits suggesting that spectral reflectance captures the endophenotypes of organisms, thereby influencing the accuracy of phenomic predictions (Rincent et al., 2018; Robert et al., 2022). Additionally, spectral reflectance can also provide estimates of plant health, disease pressure, and environmental stress (Du and Noguchi, 2017; Montesinos-López et al., 2017; Francesconi et al., 2021).

Additionally, the assessment of the DNN model across different growth stages revealed diverse accuracies, emphasizing the influence of the developmental phase of the wheat crop. Though Feekes 8 and 10 showed a higher correlation to GY, the Feekes 11 (Milky Ripe) stage exhibited the highest accuracy for GY prediction suggesting that predictive performance is not directly related to correlation. Similarly, optimal prediction stages were also observed for TW and GPC (**Supplementary Figures 3 and 4**). This highlights the need for tailored models that account for trait-specific responses during plant development. Our observation aligns with previous findings indicating varied predictive abilities of HTP-based traits like GNDVI for GY at different growth stages (Sun et al., 2019) and higher predictive power of spectral data collected near grain filling (Jackson et al., 2023). Furthermore, the observed differences in PAs across growth stages emphasize the necessity of incorporating temporal dynamics and stage-specific trait measurements into predictive models. Capturing these temporal changes can potentially improve the accuracy and robustness of DNN models for phenomic prediction in wheat breeding programs.

DNN models showed the potential in forward prediction of GY using HTP-based traits from earlier generation breeding material (PYT) evaluated at the same location (Brookings, SD) with PA of R^2^ = 0.31 (Scenario 1). However, the inclusion of multi-location data from advanced nurseries in the DNN model (Scenario 2, R² = 0.41), enhanced the PA for GY estimation in PYT by 32%. The substantial (i.e.,32%) increase in prediction accuracy highlights the value of incorporating multi-location datasets for refining GY prediction models by improving the generalizability of prediction models to predict yield across different growing conditions. This scenario also emphasizes the inherent complexities in predicting GY across diverse nurseries within a location. Robert et al. (2022) also suggested combining several spectra from different environments to increase the PA for HD, but not necessarily for GY. These results underscore the complexities involved in forward prediction modeling, especially when considering multiple nurseries within a single location or across diverse locations. However, further exploration is needed to optimize forward prediction models for various breeding scenarios.

We further evaluated the potential of using HTP-based traits in MT-GBLUP models for prediction of GY, TW, and GPC. Using ST-GBLUP as a baseline, a PA of 0.23 was achieved for GY. In contrast, the MT-GBLUP model incorporating HTP-based traits across various growth stages revealed a spectrum of improvements in PA. Notably, a 60% increase in PA for GY was observed during the booting stage (Feekes 10) as compared to ST-GBLUP (**Figure 6**). These findings align with GS investigations in animals and various crops, demonstrating improved predictive performance upon integrating correlated secondary traits (Tsuruta et al., 2011; Okeke et al., 2017). Similar improvements in GS accuracy for wheat yield prediction have been achieved using NDVI and canopy temperature as secondary traits (Crain et al., 2018; Sun et al., 2019; Sandhu et al., 2021). High heritability of secondary correlated traits, particularly when primary traits have lower heritability, contributes to the enhancement of the prediction of primary traits with the inclusion of secondary correlated HTP-based traits into multivariate GS models (Jia and Jannink, 2012; Gill et al., 2021). Therefore, optimizing the collection time for HTP-based traits may enable the breeders to maximize the selection accuracy in GS, while collecting HTP-based traits at early stages allows breeders to eliminate low-performing lines before harvest. This translates to significant savings in time and labor costs associated with managing these lines throughout the growing season.

Though we focused on evaluating the multi-trait GP for individual growth stages in the current study, another approach could use a joint analysis including the effect of growth stage in the GP using autoregressive modeling to better capture the temporal dependence of the repeated measurements over time. However, fitting a joint model with an autoregressive structure for the genetic effects matrix can be computationally challenging, especially considering the number of HTP traits involved in this study. Nevertheless, the primary objective of this study was to understand how genomic prediction performs at each growth stage for the targeted traits, and our results provided important information regarding the optimization of MT models at each growth stage. This can be particularly informative when designing breeding strategies tailored to different growth stages. However, there remains an opportunity to exploit the potential of joint models that consider temporal dependence to further improve predictive abilities for important traits.

It is worth noting that specific HTP-derived VIs such as ARI_2, GCI, and RVI_2 performed equally well or even better than all HTP-based traits combined in the MT model. This is due to the information redundancy and collinearity issues among the HTP-based traits, potentially resulting in poorer performance when using all HTP-based traits (Maimaitijiang et al., 2020). Notably, GCI and RVI_2 emerged as robust predictors for GY, while ARI_2 offered context-dependent performance enhancements. For TW prediction, ARI_2, ENDVI, and RVI_2 consistently outperformed other VIs, indicating their potential for reliable predictions with lower variability. These results emphasize the potential for exploring a wider range of HTP-derived features and VIs. By developing novel indices tailored to specific traits, researchers can leverage the rich information from HTP data to further improve accuracy and efficiency in plant breeding programs. Further research can delve deeper into the biological basis of these indices (ARI_2, GCI, RVI_2) to understand the specific plant physiological traits they represent. Additionally, investigating the generalizability of these findings across diverse environments and breeding populations will be crucial for their widespread adoption in breeding programs. Similar to our observations, several studies also found that the PA of models using Near-Infrared (NIR) spectral information was similar to or higher than models using molecular markers (Rincent et al., 2018; Krause et al., 2019; Galán et al., 2020; Zhu et al., 2021; Robert et al., 2022). Our study highlights the potential of integration of HTP-based traits in both phenomic and genomic prediction approaches, however, the choice of phenomic or genomic selection approaches will depend on specific situations in the breeding program.

## 5 Conclusion

We explored the incorporation of UAV-HTP-based traits in phenomic and genomic prediction models in predicting GY, TW, and GPC in SDSU hard winter wheat breeding. The integration of UAV multispectral remote sensing and deep learning models emerges as a promising approach for phenomic prediction of primary traits like GY, TW, and GPC. We observed superior predictive power of multitemporal multispectral data from multiple growth stages, over single-stage data. Moreover, including multilocation data over single location significantly enhanced the generalizability and robustness of the models enhancing phenotypic PA. Alternatively, the incorporation of secondary HTP-based traits in GS models demonstrates a substantial improvement in PA. Overall, this study highlights the immense potential of UAV-based phenotyping to streamline breeding programs. By providing breeders with early, comprehensive trait assessments and improved PA across environments, this approach can significantly accelerate breeding progress.

## Data availability statement

The original contributions presented in the study are included in the article/**Supplementary Material**. Further, the data supporting the conclusions of this article will be made available by the authors, without undue reservation.

## Author contributions

SK: Conceptualization, Data curation, Formal analysis, Investigation, Methodology, Software, Validation, Visualization, Writing – original draft. HG: Data curation, Formal analysis, Software, Writing – review & editing, Visualization. MB: Data curation, Software, Visualization, Writing – review & editing. SNK: Data curation, Software, Writing – review & editing. JH: Writing – review & editing, Investigation, Methodology. AB: Writing – review & editing, Investigation. PS: Writing – review & editing, Investigation, Software. GB: Resources, Writing – review & editing, Investigation. KG: Writing – review & editing. MM: Conceptualization, Data curation, Formal analysis, Investigation, Methodology, Software, Visualization, Writing – review & editing. SS: Conceptualization, Data curation, Formal analysis, Funding acquisition, Investigation, Methodology, Project administration, Resources, Software, Supervision, Validation, Visualization, Writing – original draft, Writing – review & editing.
