# Supplementary material for: Enhancing the potential of phenomic and genomic prediction in winter wheat breeding using high-throughput phenotyping and deep learning
Source: Front Plant Sci. 2024 May 30;15:1410249. doi: 10.3389/fpls.2024.1410249 (PMC11169824; doi:10.3389/fpls.2024.1410249)
Supplement: Supplementary file 1 [file DataSheet_1.pdf]

Supplementary Material

Enhancing the Potential of Phenomic and Genomic Prediction in Winter Wheat Breeding Using High-Throughput Phenotyping and Deep Learning

Swas Kaushal[1], Harsimardeep S Gill[1], Mohammad Maruf Billah[2], Shahid Nawaz Khan[2], Jyotirmoy Halder[1], Amy Bernardo[3], Paul St. Amand[3], Guihua Bai[3], Karl Glover[1], Maitiniyazi Maimaitijiang[2*], Sunish K. Sehgal[1*]

[1]Department of Agronomy, Horticulture and Plant Science, South Dakota State University, Brookings, SD, United States

[2]Department of Geography & Geospatial Sciences, Geospatial Sciences Center of Excellence, South Dakota State University, Brookings, SD, United States

[3]USDA-ARS, Hard Winter Wheat Genetics Research Unit, Manhattan, KS, United States

Supplementary Tables and Figures

Supplementary Table 1: List of vegetation indices and equations extracted from multispectral images used in this study.

Spectral feature name	Formulation	Ref.
5 original reflectance bands: Blue (B), Green (G), Red (R), Red edge (RE), Near Infrared (NIR)	The reflectance value of each band	-
Normalized difference vegetation index	NDVI = (NIR – R) / (NIR+ R)	(Rouse Jr et al., 1974)
Green Normalized Difference Vegetation Index (GNDVI)	GNDVI = (NIR-G)/(NIR+G)	(Gitelson & Merzlyak, 1997)
Ratio Vegetation Index 1	RVI_1 = NIR/R	(Richardson & Wiegand, 1977)
Green Chlorophyll Index	GCI = (NIR/G)-1.0	(Gitelson et al.,
Rice Growth Vegetation Index	RGVI = R/G	(Nuarsa et al.,
Difference Vegetation Index	DVI = NIR-R	(Tucker, 1979)
Soil-Adjusted Vegetation	SAVI = ((NIR-R)/(NIR+R+L))*(1.0+L), L =	(Huete, 1988)
Modified Soil Adjusted Vegetation Index	MSAVI=0.5*((2.0*NIR)+1.0-np.sqrt(np.square(2.0*NIR+1.0)-	(Qi et al., 1994)
Optimized Soil Adjusted Vegetation Index	OSAVI = (NIR-R)/(NIR+R+0.16)	(Rondeaux et al., 1996)
Renormalized Difference Vegetation Index	RDVI = np.sqrt((np.square(NIR-R))/(NIR+R))	(Roujean & Breon, 1995)
Triangular Vegetation Index	TVI = 60.0*(NIR-G)-100.0*(R-G)	(Deering, 1975)
Transformed Soil Adjusted Vegetation Index	TSAVI = (a*(NIR-a*R-b))/(a*NIR+R-a*b), a, b = 0.96916, 0.084726	(Baret & Guyot, 1991)
Perpendicular Vegetation Index	PVI = (NIR-a*R-b)/np.sqrt(1+np.square(a)), a, b =	(Richardson & Wiegand, 1977)
Soil-Adjusted Vegetation Index 2	SAVI_2 = NIR/(R-(b/a)), a, b = 0.96916, 0.084726	(Huete, 1988)
Adjusted Transformed Soil Adjusted Vegetation Index	ATSAVI = (a*(-a*R-b))/(a*NIR+R-a*b+X*(1+np.square(a))), a, b, X = 0.96916, 0.084726, 0.08	(Baret & Guyot, 1991)
Normalized Difference Water Index	NDWI = (G-NIR)/(G+NIR)	(McFeeters, 1996)
Kawashima index	IKAW = (R -B)/(R+B)	(Kawashima & Nakatani, 1998)
Simple Ratio Pigment Index	SRPI = B/R	(Peñuelas et al., 1995)

Ratio Vegetation Index 2	RVI_2 = NIR/G	(Pearson & Miller, [illegible]
Modified Chlorophyll Absorption in Reflective	MCARI = (RE-R-0.2*(RE-G))*(RE/R)	(Daughtry et al., 2000)
Modified Chlorophyll Absorption in Reflective	MCARI_1 = 1.2*(2.5*(NIR-R)-1.3*(NIR-G))	(Haboudane et al., 2004)
Modified Chlorophyll Absorption in Reflective	MCARI_2 = 1.5*(2.5*(NIR-R)-1.3*(NIR-G))*(np.square(2.0*NIR+1))-(6.0*NIR-	(Haboudane et al., 2004)
Modified Triangular Vegetation Index	MTVI_1 = 1.2*(1.2*(NIR-G)-2.5*(R-G))	(Haboudane et al., 2004)
Modified Triangular Vegetation Index 2	MTVI_2 = 1.5*(1.2*(NIR-G)-2.5*(R-G))*(np.square(2*NIR+1))-(6.0*NIR-	(Haboudane et al., 2004)
Modified chlorophyll absorption ratio index/Second modified	R_MCARI_MTVI2 = ((RE-R-0.2*(RE-G))*(RE/R))/(1.5*(1.2*(NIR-G)-2.5*(R-G))*(np.square(2*NIR+1))-(6.0*NIR-	(Eitel et al., 2007)
Enhanced Vegetation Index	EVI = (NIR-R)/(NIR+6.0*R-7.5*B+1.0)	(Huete et al., 1997)
Datt Index	DATT = (NIR-RE)/(NIR-R)	(Datt, 1999)
Double-peak Canopy Nitrogen Index	DNCI = (RE-G)/(RE+G)	(Chen et al., 2010)
Plant Senescence Reflectance Index	PSRI = (R-G)/RE	(Merzlyak et al., 1999)
Structure Intensive Pigment Vegetation Index	SIPI = (NIR-B)/(NIR+R)	(Peñuelas et al., 1995)
Spectral Polygon Vegetation Index	SPVI = 0.4*3.7*(NIR-R)-1.2*np.absolute(G-R)	(Vincini et al., 2006)
Transformed Chlorophyll Absorption Reflectance Index	TCARI = 3.0*((RE-R)-0.2*(RE-G)*(RE/R))	(Haboudane et al., 2002)
Transformed Chlorophyll Absorption in the Reflectance Index/Optimized Soil-Adjusted Vegetation Index	R_TCARI_OSAVI = (3.0*((RE-R)-0.2*(RE-G)*(RE/R)))/((NIR-R)/(NIR+R+0.16))	(Haboudane et al., 2002)
Red edge relative index	RERI = (RE-R)/NIR	(Haboudane et [illegible]
Normalized difference red edge index	NDRE = (NIR-RE)/(NIR+RE)	(Fitzgerald et al., 2010)
MERIS Terrestrial Chlorophyll Index	MTCI = (NIR-RE)/(RE-R)	(Dash & Curran, 2004)
Enhanced Vegetation Index 2	EVI_2 = 2.5*((NIR-R)/(NIR+2.4*R+1.0))	(Huete et al., 1997)
Red-Edge Chlorophyll Index	RECI = (NIR/RE)-1	(Haboudane et al., 2002)

Normalized excess green vegetation index	NEXG = (2*G-R-B)/(G+R+B)	(Woebbecke et al., 1995)
Normalized green–red	NGRDI = (G-R)/(G+R)	(Tucker, 1979)
Enhanced Normalized Difference Vegetation Index	ENDVI = (NIR+G-2.0*B)/(NIR+G+2.0*B)	(Anchal et al., 2022)
Anthocyanin Reflectance Index 2	ARI_2 = NIR*(1.0/G)-(1.0/RE))	(Gitelson et al., 2001)
Carotenoid Reflectance Index 2	CRI_2 = (1.0/G)-(1.0/RE)	(Gitelson et al., 2003)

Supplementary Table 2. Summary statistics and broad-sense heritability (H^2) of recorded agronomic traits.

Trait	Mean	H^2
Grain Yield (GY)(kg/ha)	70.30	0.36
Grain Protein (GP)(%)	12.48	0.26
Test Weight (TW)(g/l)	58.88	0.87

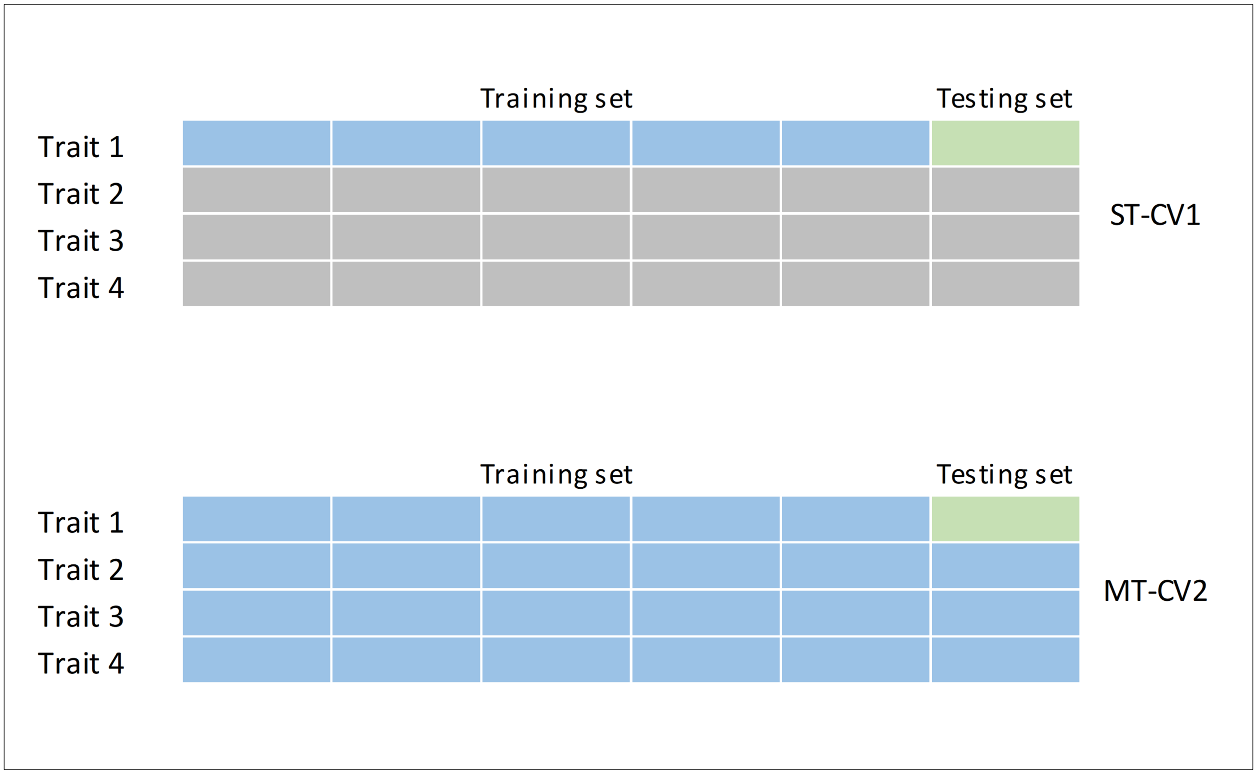

Supplementary Figure 1. Prediction accuracies were estimated for the single-trait (ST) model with cross-validation scheme 1 (ST-CV1) and multi-trait (MT) model with cross-validation scheme 2 (MT-CV2).

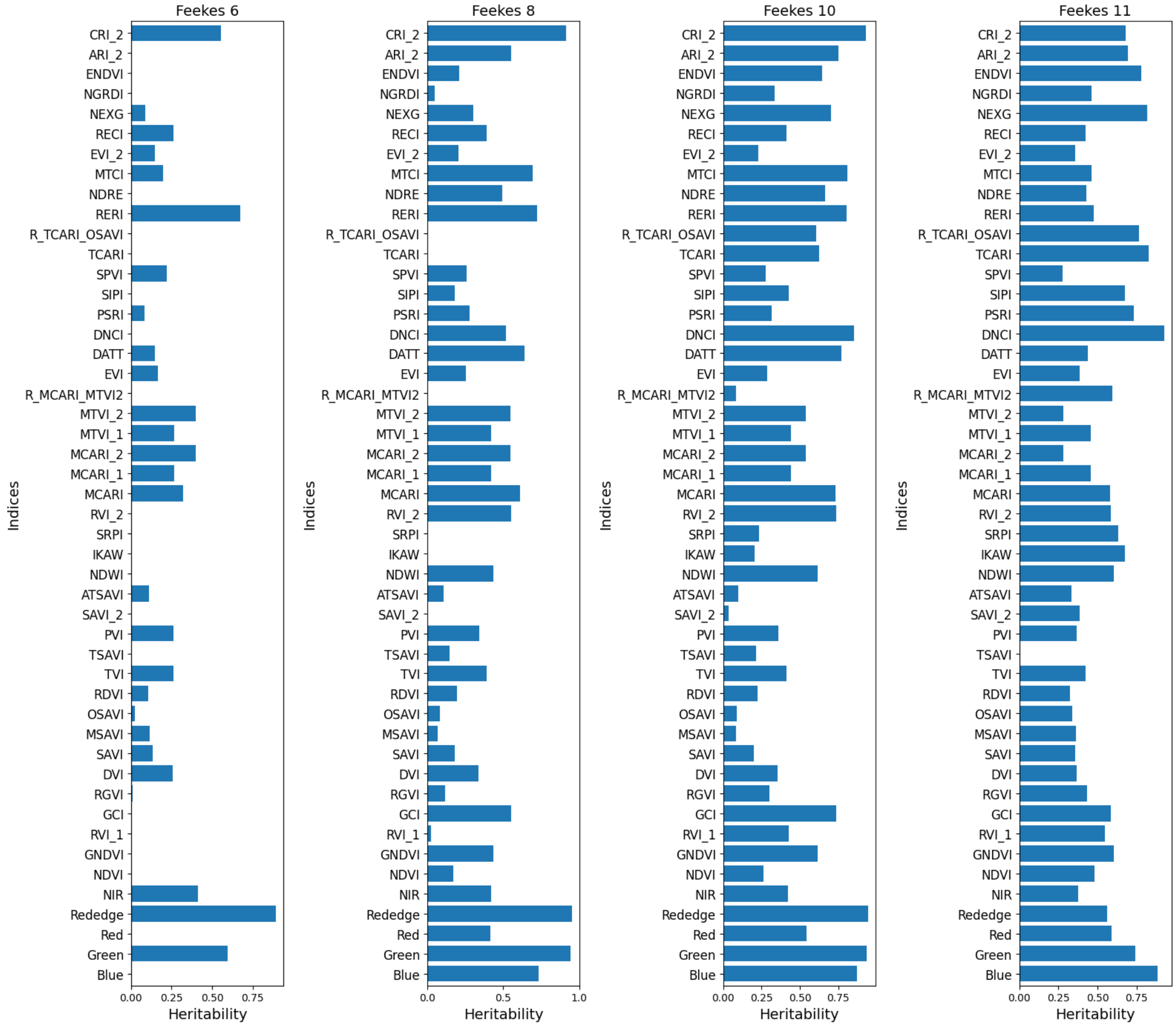

Supplementary Figure 2. Broad-sense heritability (H^2) of High-Throughput Phenotyping (HTP) based traits across various growth stages (Feekes 6: jointing, Feekes 8: Flag leaf, Feekes 10: Booting, Feekes 11: Milk ripe).

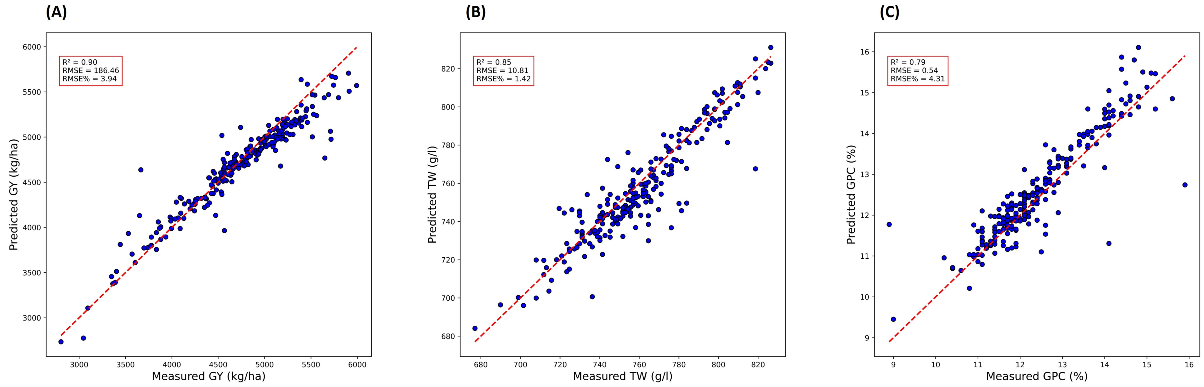

Supplementary Figure 3. Phenomic predictions for grain yield (GY), B) test weight (TW), and C) grain protein (GPC), using DNN with combined High-Throughput Phenotyping (HTP) based traits on the training set (70%) for ELITE and AYT at Brookings.

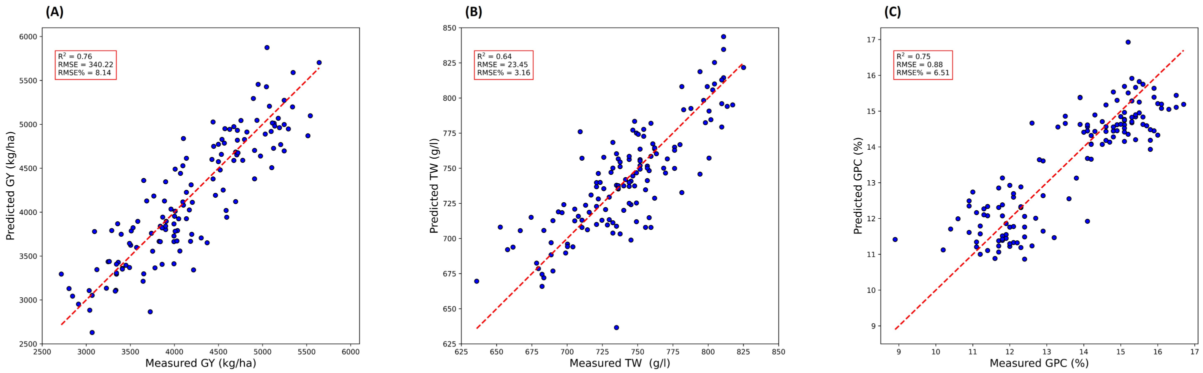

Supplementary Figure 4. A) Phenomic predictions for grain yield (GY), B) Test weight (TW), and C) grain protein content (GPC). Predictions were made on the testing set (30%) using DNN trained on ELITE and AYT at Brookings and Dakota Lakes (multi-locations).

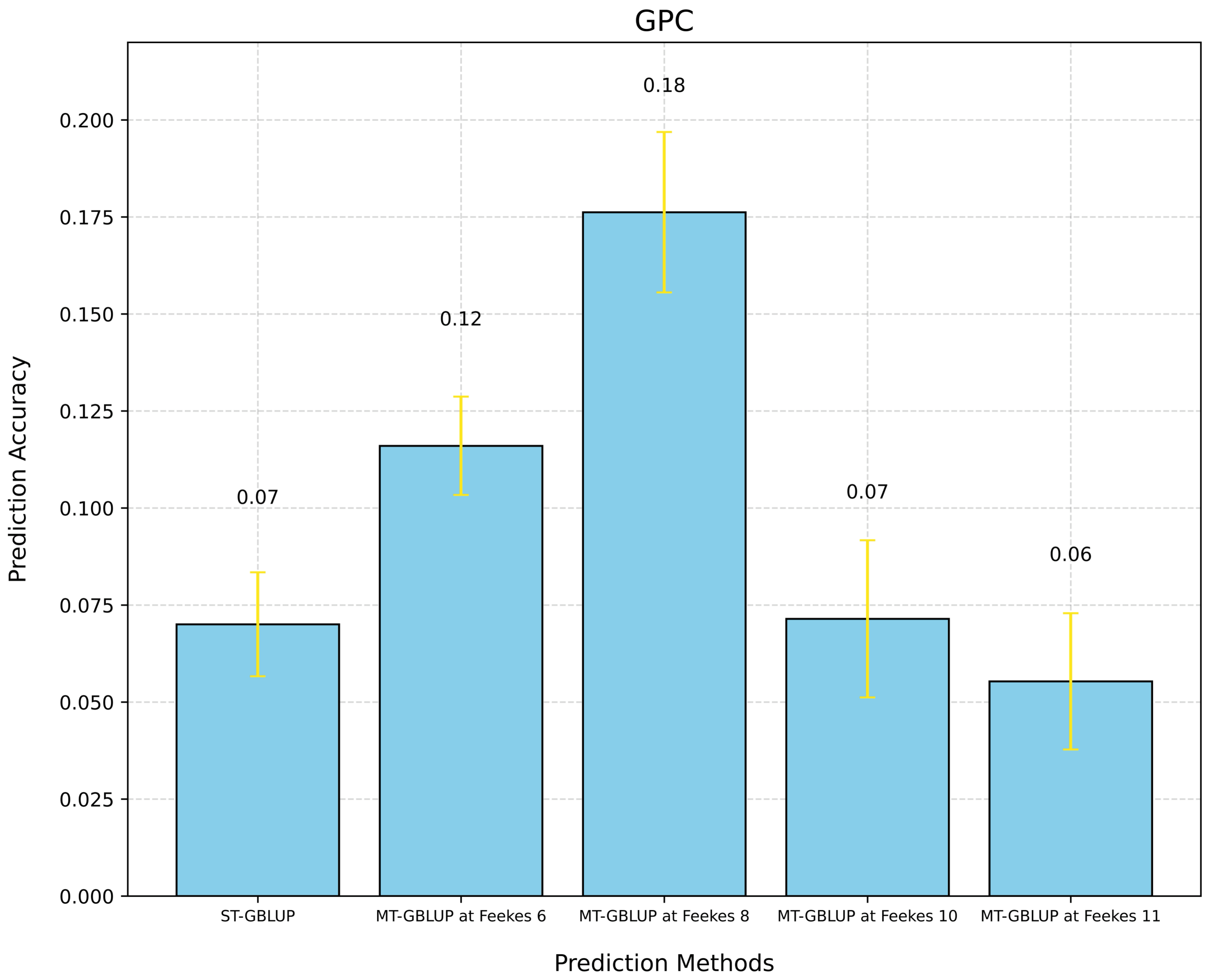

Supplementary Figure 5. Comparison of Prediction accuracy for Grain Protein (GPC) using Single-Trait GBLUP (ST-GBLUP) with cross-validation scheme ST-CV1 and Multi-Trait GBLUP (MT-GBLUP) incorporating High-Throughput Phenotyping (HTP) based traits with cross-validation scheme MT-CV2 across different growth stages (Feekes 6: jointing, Feekes 8: Flag leaf, Feekes 10: Booting, Feekes 11: Milk ripe).

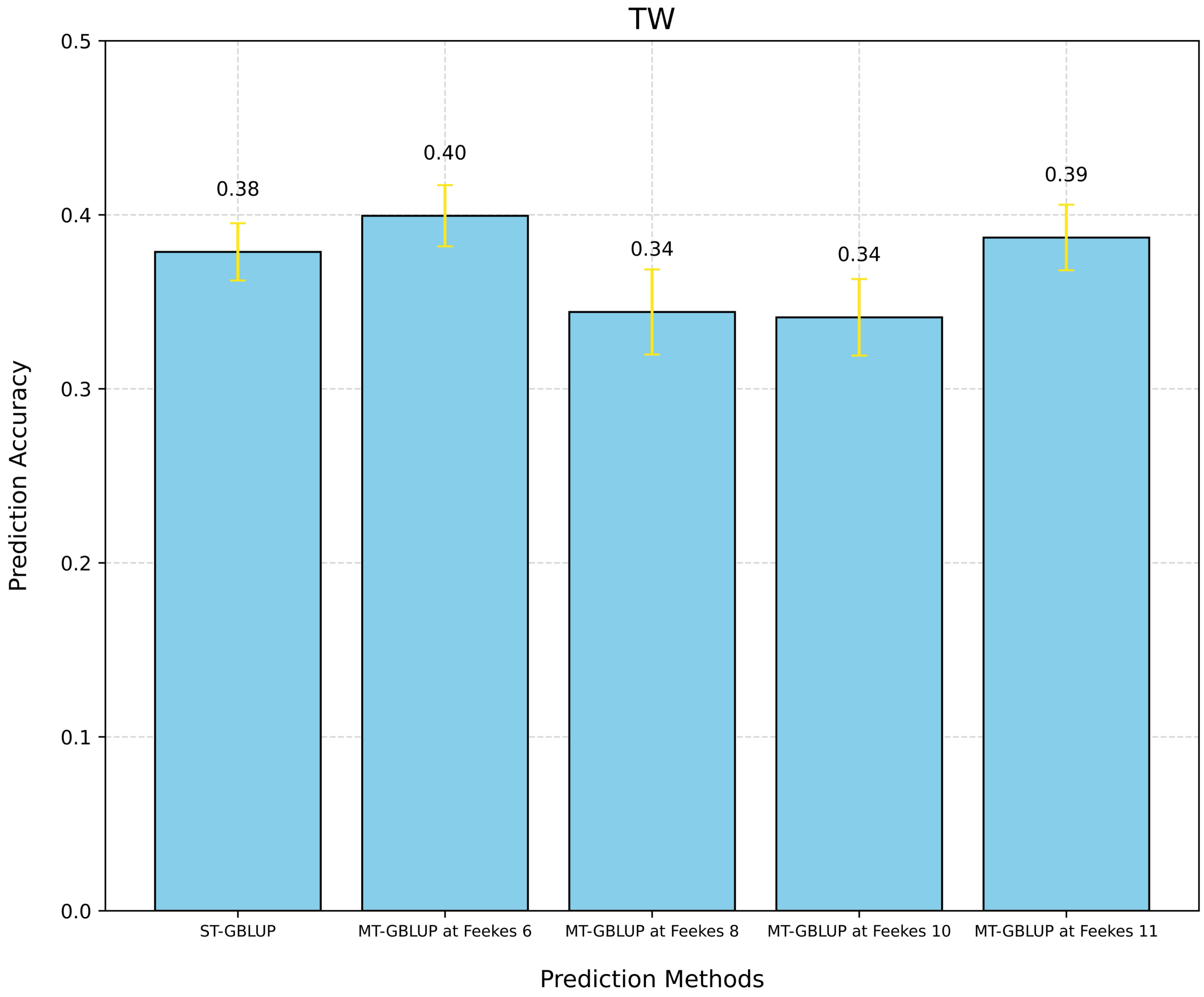

Supplementary Figure 6. Comparison of Prediction accuracy for Test Weight (TW) using Single-Trait GBLUP (ST-GBLUP) with cross-validation scheme ST-CV1 and Multi-Trait GBLUP (MT-GBLUP) High-Throughput Phenotyping (HTP) based traits with cross-validation scheme MT-CV2 across different growth stages (Feekes 6: jointing, Feekes 8: Flag leaf, Feekes 10: Booting, Feekes 11: Milk ripe).

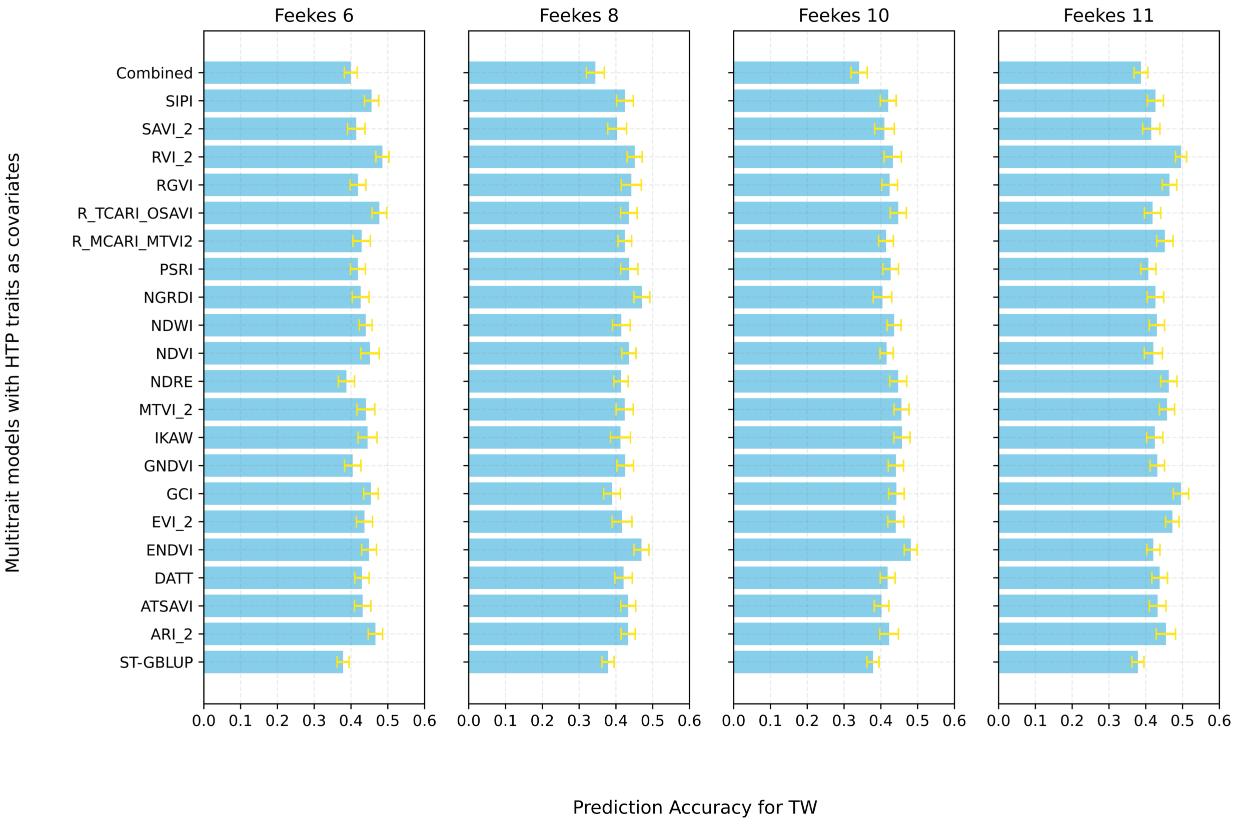

Supplementary Figure 7. Prediction accuracy for Test Weight (TW) using Single-Trait GBLUP (ST-GBLUP) with cross-validation scheme ST-CV1 and Multi-Trait GBLUP (MT-GBLUP) incorporating High-Throughput Phenotyping (HTP) based traits with cross-validation scheme MT-CV2 across different growth stages different growth stages (Feekes 6: jointing, Feekes 8: Flag leaf, Feekes 10: Booting, Feekes 11: Milk ripe).

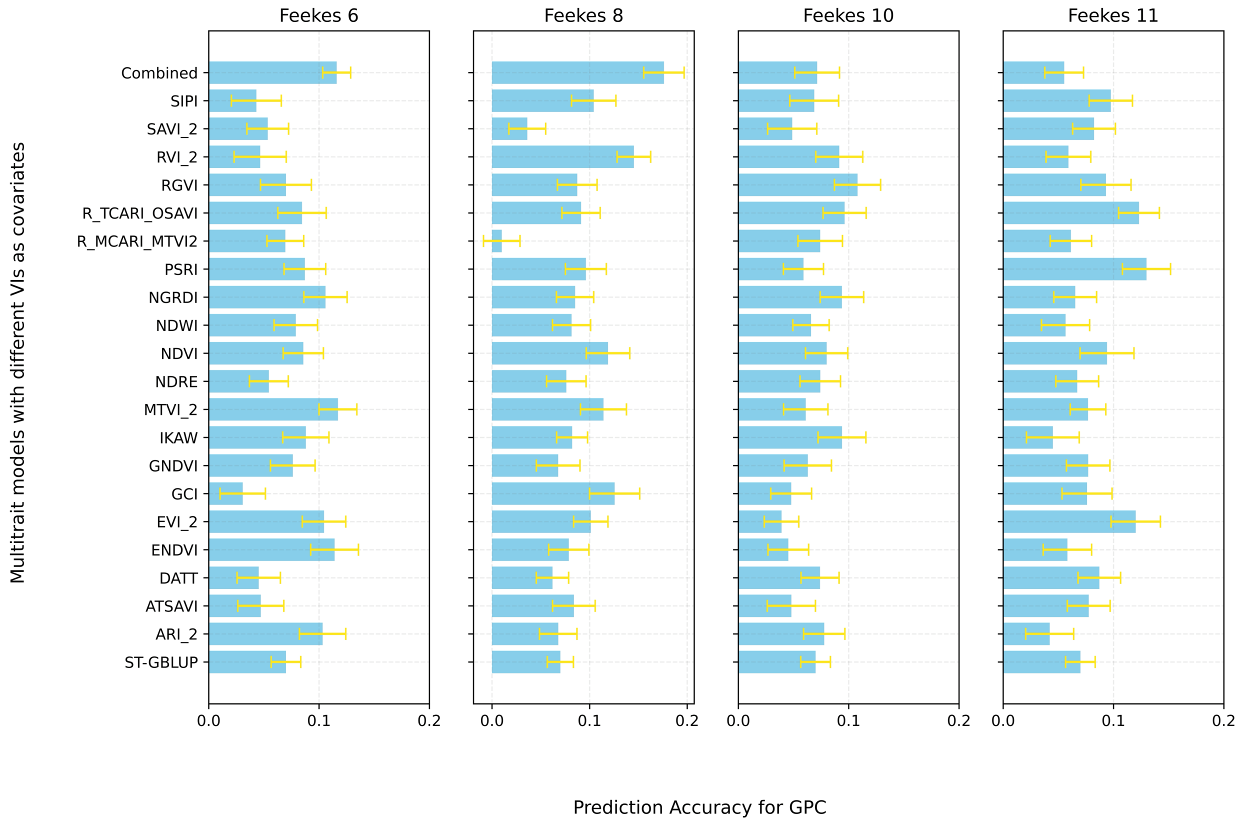

Supplementary Figure 8. Prediction accuracy for Grain Protein (GPC) using Single-Trait GBLUP (ST-GBLUP) with cross-validation scheme ST-CV1 and Multi-Trait GBLUP (MT-GBLUP) incorporating High-Throughput Phenotyping (HTP) based traits with cross-validation scheme MT-CV2 across different growth stages different growth stages (Feekes 6: jointing, Feekes 8: Flag leaf, Feekes 10: Booting, Feekes 11: Milk ripe).

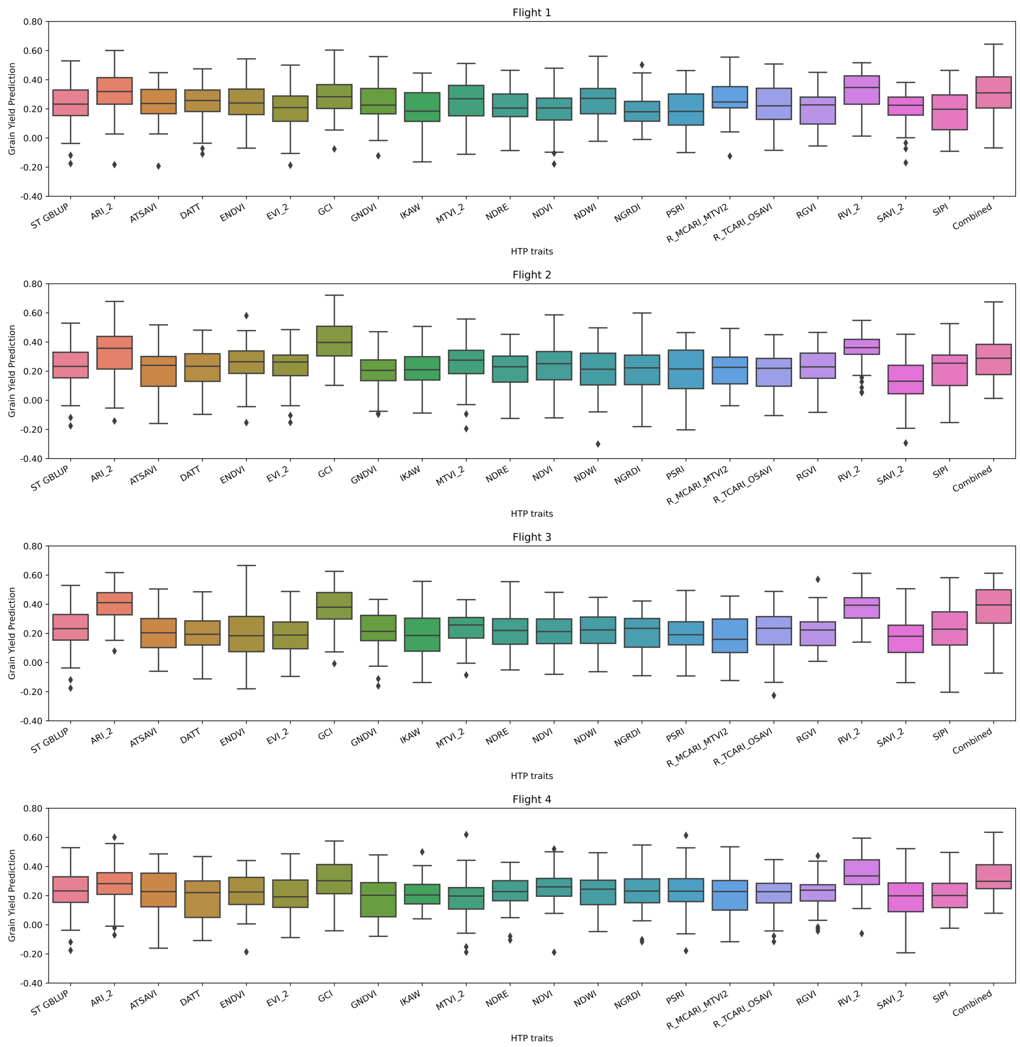

Supplementary Figure 9. Distribution of prediction accuracy estimates for Grain Yield (GY) across multiple repetitions using Single-Trait GBLUP (ST-GBLUP) with cross-validation scheme ST-CV1 and Multi-Trait GBLUP (MT-GBLUP) incorporating High-Throughput Phenotyping (HTP) based traits with cross-validation scheme MT-CV2 across flights. Each boxplot captures the variation in prediction accuracy across replicates for different prediction models.

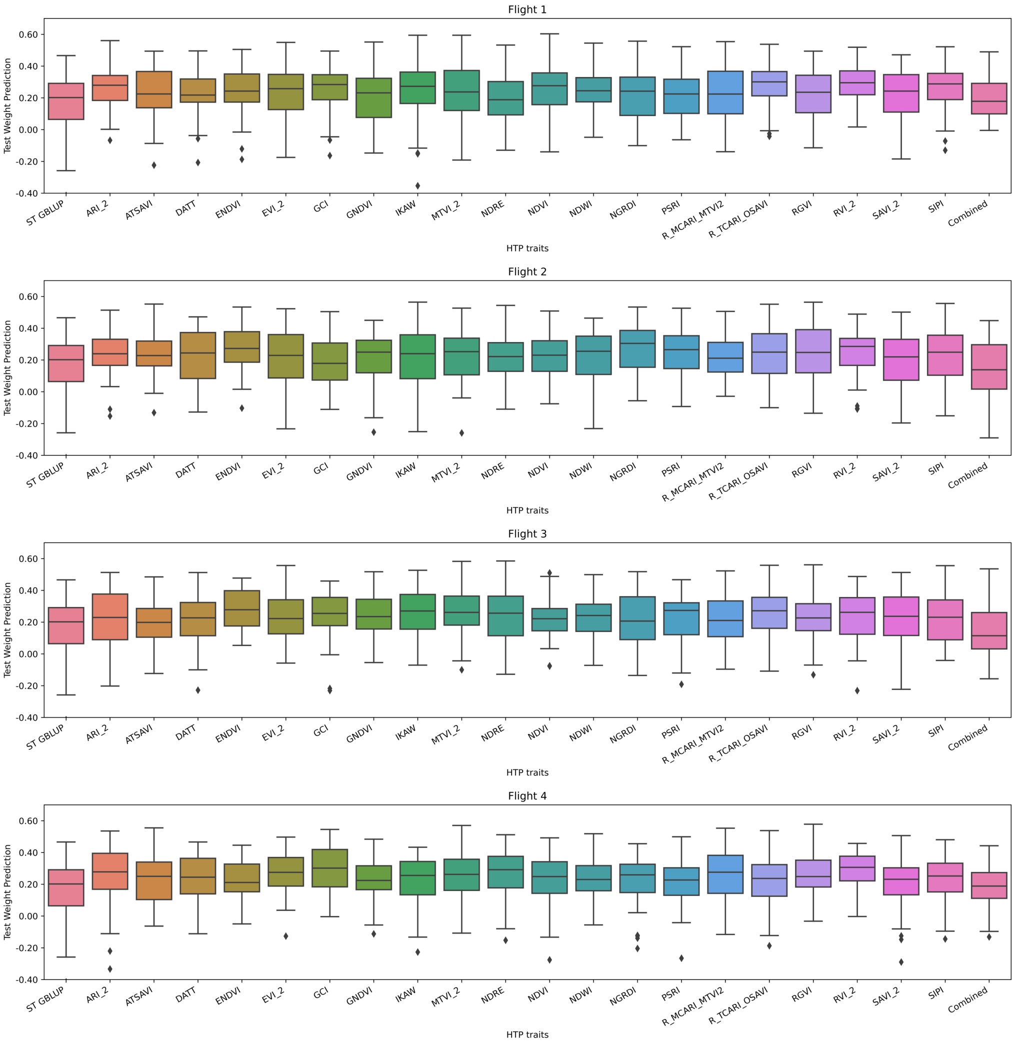

Supplementary Figure 10. Distribution of prediction accuracy estimates for Test Weight (TW) across multiple repetitions using Single-Trait GBLUP (ST-GBLUP) with cross-validation scheme ST-CV1 and Multi-Trait GBLUP (MT-GBLUP) incorporating High-Throughput Phenotyping (HTP) based traits with cross-validation scheme MT-CV2 across flights. Each boxplot captures the variation in prediction accuracy across replicates for different prediction models.

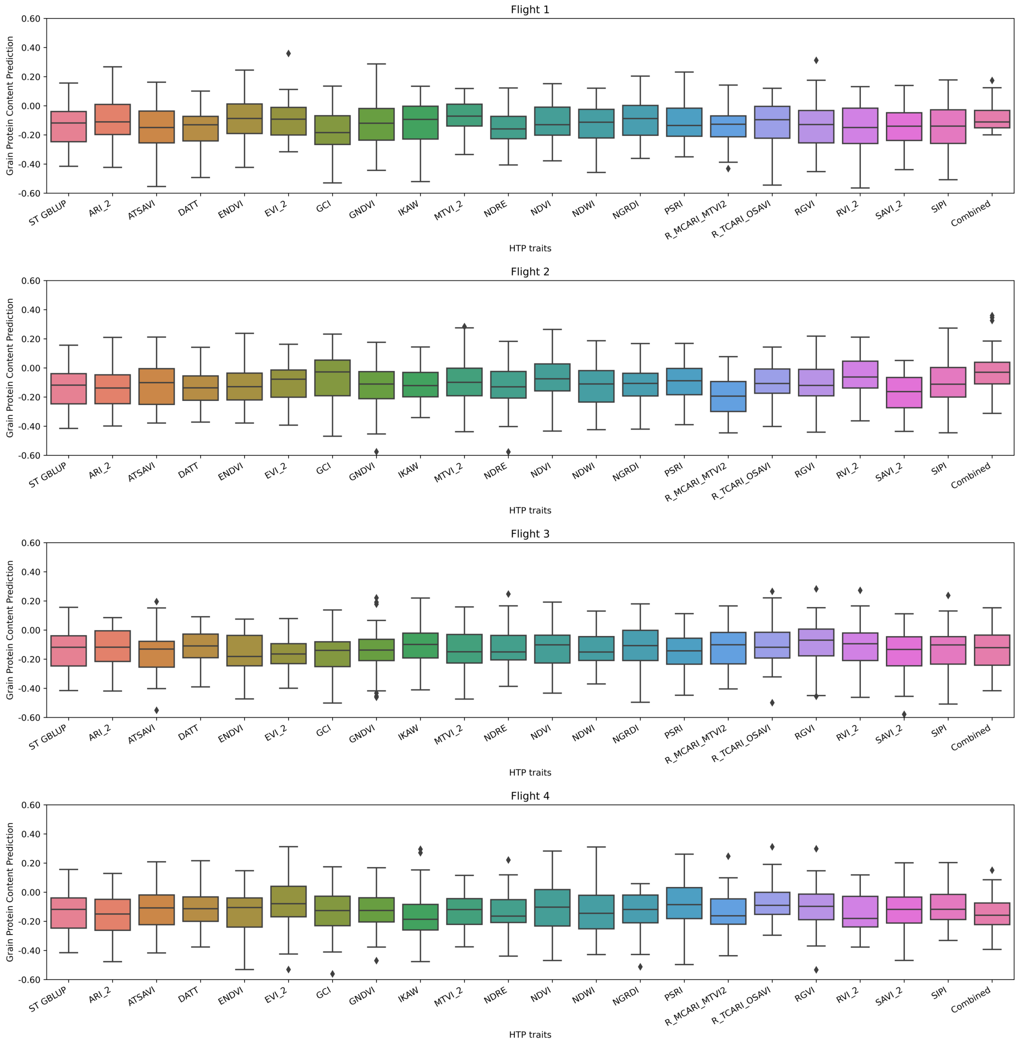

Supplementary Figure 11. Distribution of prediction accuracy estimates for Grain Protein Content (GPC) across multiple repetitions using Single-Trait GBLUP (ST-GBLUP) with cross-validation scheme ST-CV1 and Multi-Trait GBLUP (MT-GBLUP) incorporating High-Throughput Phenotyping (HTP) based traits with cross-validation scheme MT-CV2 across flights. Each boxplot captures the variation in prediction accuracy across replicates for different prediction models.

REFERENCES

Anchal, S., Bahuguna, S., Priti, Pal, P. K., Kumar, D., Murthy, P. S., & Kumar, A. (2022). Non-destructive method of biomass and nitrogen (N) level estimation in Stevia rebaudiana using various multispectral indices. *Geocarto International*, *37*(22), 6409-6421.

Baret, F., & Guyot, G. (1991). Potentials and limits of vegetation indices for LAI and APAR assessment. *Remote sensing of environment*, *35*(2-3), 161-173.

Chen, P., Haboudane, D., Tremblay, N., Wang, J., Vigneault, P., & Li, B. (2010). New spectral indicator assessing the efficiency of crop nitrogen treatment in corn and wheat. *Remote sensing of environment*, *114*(9), 1987-1997.

Dash, J., & Curran, P. (2004). The MERIS terrestrial chlorophyll index.

Datt, B. (1999). A new reflectance index for remote sensing of chlorophyll content in higher plants: tests using Eucalyptus leaves. *Journal of plant physiology*, *154*(1), 30-36.

Daughtry, C. S., Walthall, C., Kim, M., De Colstoun, E. B., & McMurtrey Iii, J. (2000). Estimating corn leaf chlorophyll concentration from leaf and canopy reflectance. *Remote sensing of environment*, *74*(2), 229-239.

Deering, D. (1975). Measuring" forage production" of grazing units from Landsat MSS data. Proceedings of the Tenth International Symposium of Remote Sensing of the Envrionment,

Eitel, J., Long, D., Gessler, P., & Smith, A. (2007). Using in-situ measurements to evaluate the new RapidEye™ satellite series for prediction of wheat nitrogen status. *International Journal of Remote Sensing*, *28*(18), 4183-4190.

Fitzgerald, G., Rodriguez, D., & O'Leary, G. (2010). Measuring and predicting canopy nitrogen nutrition in wheat using a spectral index—The canopy chlorophyll content index (CCCI). *Field crops research*, *116*(3), 318-324.

Gitelson, A. A., Gritz, Y., & Merzlyak, M. N. (2003). Relationships between leaf chlorophyll content and spectral reflectance and algorithms for non-destructive chlorophyll assessment in higher plant leaves. *Journal of plant physiology*, *160*(3), 271-282.

Gitelson, A. A., & Merzlyak, M. N. (1997). Remote estimation of chlorophyll content in higher plant leaves. *International Journal of Remote Sensing*, *18*(12), 2691-2697.

Gitelson, A. A., Merzlyak, M. N., & Chivkunova, O. B. (2001). Optical properties and nondestructive estimation of anthocyanin content in plant leaves¶. *Photochemistry and photobiology*, *74*(1), 38-45.

Haboudane, D., Miller, J. R., Pattey, E., Zarco-Tejada, P. J., & Strachan, I. B. (2004). Hyperspectral vegetation indices and novel algorithms for predicting green LAI of crop canopies: Modeling and validation in the context of precision agriculture. *Remote sensing of environment*, *90*(3), 337-352.

Haboudane, D., Miller, J. R., Tremblay, N., Zarco-Tejada, P. J., & Dextraze, L. (2002). Integrated narrow-band vegetation indices for prediction of crop chlorophyll content for application to precision agriculture. *Remote sensing of environment*, *81*(2-3), 416-426.

Huete, A., Liu, H., Batchily, K., & Van Leeuwen, W. (1997). A comparison of vegetation indices over a global set of TM images for EOS-MODIS. *Remote sensing of environment*, *59*(3), 440-451.

Huete, A. R. (1988). A soil-adjusted vegetation index (SAVI). *Remote sensing of environment*, *25*(3), 295-309.

Kawashima, S., & Nakatani, M. (1998). An algorithm for estimating chlorophyll content in leaves using a video camera. *Annals of Botany*, *81*(1), 49-54.

McFeeters, S. K. (1996). The use of the Normalized Difference Water Index (NDWI) in the delineation of open water features. *International Journal of Remote Sensing*, *17*(7), 1425-1432.
Merzlyak, M. N., Gitelson, A. A., Chivkunova, O. B., & Rakitin, V. Y. (1999). Non-destructive optical detection of pigment changes during leaf senescence and fruit ripening. *Physiologia plantarum*, *106*(1), 135-141.
Nuarsa, I. W., Nishio, F., & Hongo, C. (2011). Relationship between rice spectral and rice yield using MODIS data. *Journal of Agricultural Science*, *3*(2), 80.
Pearson, R. L., & Miller, L. D. (1972). Remote mapping of standing crop biomass for estimation of the productivity of the shortgrass prairie. *Remote sensing of environment, VIII*, 1355.
Peñuelas, J., Filella, I., & Gamon, J. A. (1995). Assessment of photosynthetic radiation-use efficiency with spectral reflectance. *New Phytologist*, *131*(3), 291-296.
Qi, J., Chehbouni, A., Huete, A. R., Kerr, Y. H., & Sorooshian, S. (1994). A modified soil adjusted vegetation index. *Remote sensing of environment*, *48*(2), 119-126.
Richardson, A. J., & Wiegand, C. (1977). Distinguishing vegetation from soil background information. *Photogrammetric engineering and remote sensing*, *43*(12), 1541-1552.
Rondeaux, G., Steven, M., & Baret, F. (1996). Optimization of soil-adjusted vegetation indices. *Remote sensing of environment*, *55*(2), 95-107.
Roujean, J.-L., & Breon, F.-M. (1995). Estimating PAR absorbed by vegetation from bidirectional reflectance measurements. *Remote sensing of environment*, *51*(3), 375-384.
Rouse Jr, J., Haas, R. H., Deering, D., Schell, J., & Harlan, J. C. (1974). *Monitoring the vernal advancement and retrogradation (green wave effect) of natural vegetation*.
Tucker, C. J. (1979). Red and photographic infrared linear combinations for monitoring vegetation. *Remote sensing of environment*, *8*(2), 127-150.
Vincini, M., Frazzi, E., & D'Alessio, P. (2006). Angular dependence of maize and sugar beet VIs from directional CHRIS/Proba data. Proc. 4th ESA CHRIS PROBA Workshop,
Woebbecke, D. M., Meyer, G. E., Von Bargen, K., & Mortensen, D. A. (1995). Color indices for weed identification under various soil, residue, and lighting conditions. *Transactions of the ASAE*, *38*(1), 259-269.
